# Random Forests Based Group Importance Scores and Their Statistical Interpretation: Application for Alzheimer's Disease

**DOI:** 10.3389/fnins.2018.00411

**Published:** 2018-06-29

**Authors:** Marie Wehenkel, Antonio Sutera, Christine Bastin, Pierre Geurts, Christophe Phillips

**Affiliations:** ^1^Department of Computer Science and Electrical Engineering, Montefiore Institute, University of Liège, Liège, Belgium; ^2^GIGA-CRC in silico Medicine, University of Liège, Liège, Belgium; ^3^GIGA-CRC in vivo Imaging, University of Liège, Liège, Belgium

**Keywords:** machine learning, random forests, Alzheimer's disease, feature selection, group-based method, prognosis system, FDG-PET

## Abstract

Machine learning approaches have been increasingly used in the neuroimaging field for the design of computer-aided diagnosis systems. In this paper, we focus on the ability of these methods to provide interpretable information about the brain regions that are the most informative about the disease or condition of interest. In particular, we investigate the benefit of group-based, instead of voxel-based, analyses in the context of Random Forests. Assuming a prior division of the voxels into non overlapping groups (defined by an atlas), we propose several procedures to derive group importances from individual voxel importances derived from Random Forests models. We then adapt several permutation schemes to turn group importance scores into more interpretable statistical scores that allow to determine the truly relevant groups in the importance rankings. The good behaviour of these methods is first assessed on artificial datasets. Then, they are applied on our own dataset of FDG-PET scans to identify the brain regions involved in the prognosis of Alzheimer's disease.

## 1. Introduction

Alzheimer's disease is currently the neurodegenerative disease the most often encountered in aged population and, as the world's population ages, the prevalence of the disease is expected to increase (Brookmeyer et al., 2007). Much research has been undertaken in order to find treatments to delay the onset of the disease or slow down its progress (Hardy and Selkoe, 2002; Roberson and Mucke, 2006). As current clinical trials testing amyloid-modifying therapies in demented individuals failed to show any effect, it is believed that interventions must start before the onset of clinical symptoms (Sperling et al., 2014). Nervertheless, it still remains a challenge to predict if one individual will develop the disease before brain damages and irreversible symptoms have already appeared. Before a definitive AD diagnosis has been established clinically with neuropsychological tests, individuals go through a stage of “mild cognitive impairment” (MCI) during which predicting the outcome, stabilisation or worsening of the cognitive deficit, is difficult. Many studies have focused on this prodromal stage of Alzheimer's disease (Petersen et al., 1999, 2001).

Machine learning (ML) methods have been increasingly used over the years in neuroimaging in general and in particular also for the design of prognosis systems for Alzheimer's disease (see Rathore et al., 2017 for a review of classification frameworks designed for AD and its prodromal stages). While structural magnetic resonance imaging (sMRI) modality is helpful to detect brain atrophy from MCI to AD (Jack et al., 1999; Killiany et al., 2000), functional MRI and fluorodeoxyglucose positron-emission tomography (FDG-PET) highlight function and metabolism alterations of the brain (Chételat et al., 2003; Rombouts et al., 2005). Researchers exploit these information with machine learning algorithms to achieve the best possible predictive performance or sometimes to learn more about the brain areas involved in the studied disease. Due to high dimensionality issues, it is often necessary to use feature reduction methods before the learning process in order to improve performance (Chu et al., 2012; Segovia et al., 2012; Mwangi et al., 2014). Feature selection presents in general the benefit of keeping the results interpretable, unlike feature extraction methods such as partial least squares (Wold et al., 1984; Geladi and Kowalski, 1986) or principal component analysis (Jolliffe, 1986).

One of the most commonly used ML methods in neuroimaging is Support Vector Machines (SVM) (Hearst et al., 1998). The success of this method in this domain is due to its competitive performance when the number of features is large in comparison with the number of samples. In addition, when exploited with linear kernels, SVM provide weights for each voxel enabling the visualisation of brain patterns linked to the diagnosis (Vemuri et al., 2008; Zhang et al., 2011). Nevertheless, these methods typically use the whole set of voxels to compute a prediction and, so, it is difficult to threshold the weights and interpret them in terms of their role importance in the patient condition. Sparsity-enforcing linear methods, such as Lasso or Elastic-net (Tibshirani, 1996; Zou and Hastie, 2005), are alternative techniques that embed a more explicit feature selection mechanism through a L1-penalization of the weight vector. These methods have been used with some success to analyse neuro-imaging data (Carroll et al., 2009; Ryali et al., 2010; Casanova et al., 2011). Tree-based ensemble methods, such as Random Forests or Extremely Randomised Trees (Breiman, 2001; Geurts et al., 2006), are also known for their good predictive performance in high-dimensional/small sample size settings and furthermore provide interpretable results through feature importance scores. Their non-parametric nature makes them an interesting alternative to linear methods. Although they have not been studied extensively in the neuroimaging community, there is evidence in the literature of their potential in such applications (Kuncheva et al., 2010; Langs et al., 2011; Gray et al., 2013; Ganz et al., 2015; Wehenkel et al., 2017).

When it comes to highlight brain regions involved in the studied disease, the main benefit of the aforementioned ML methods is their multivariate and non-parametric (for trees) nature, which potentially allows them to detect complex patterns in the data. Unlike statistical tests however, which associate to each problem feature a (corrected) *p*-value, scores extracted from ML methods, such as SVM weights and RF feature importances, can not be interpreted as easily. This makes very difficult the determination of a score threshold to distinguish the truly relevant features from the irrelevant ones in the resulting multivariate rankings. To circumvent this issue, the predictive performance of a ML model trained on a subset of features is therefore often used as a proxy to evaluate the relevance of the features in this subset and can be used to guide the search for the truly relevant features. For example, the regularisation level, and thus the sparsity, of sparse linear models can be tuned using cross-validation. Recursive feature elimination (Guyon et al., 2002; Guyon and Elisseeff, 2003) is an efficient procedure to find an optimal subset of features from SVM. A first SVM model is used to ranked all features. The lowest ranked features are then removed, a new model is retrained to rank the remaining features, and the process is repeated until no features are left. The feature subset that minimises cross-validation error in the resulting nested sequence is returned as the final optimal feature subset. In the context of Random Forests, Ganz et al. (2015) have proposed instead to remove iteratively the top ranked features and stop when the performance obtained on the remaining features is not better than random. While efficient mainly as a way to improve predictive performance, these methods do not really provide interpretable scores and, since cross-validation error is only a proxy for feature relevance, there is still a risk with these methods to either miss features or to select irrelevant ones (Huynh-Thu et al., 2012).

An alternative approach, proposed by several authors (Ge et al., 2003; Mourão-Miranda et al., 2005; Klöppel et al., 2008; Altmann et al., 2010; Huynh-Thu et al., 2012), is to exploit permutation tests in order to replace ML based scores by *p*-values like scores that are more interpretable and can be more easily thresholded. The general idea of these methods is to try to estimate for each score value *v* either the proportion of irrelevant features among those that have obtained a score higher than *v* (false discovery rate, FDR) or the probability that an irrelevant feature can reach such a high score (family-wise error rate, FWER). These values are estimated by exploiting more or less sophisticated permutation schemes that simulate feature irrelevance by randomly shuffling the labels. In order not to overestimate FDR or FWER values, these permutation schemes have to take into account the dependence that inevitably exists between importance scores derived from multivariate ML methods. Huynh-Thu et al. (2012) provide an empirical comparison of several of these methods, notably applied on RF importance scores, in the context of microarray classification problems in bioinformatics.

While very good results can be obtained by applying ML methods on neuroimaging data, identifying relevant features among hundreds of thousands of voxels with permutation tests is expected to be very challenging both computationally and statistically (as the more features, the higher the estimated FDR or FWER, because of multiple testing issues). In addition, the interpretability of a selection or ranking at the level of voxels is questionable. Because of the high expected spatial correlation among voxels, it is very likely than neighbouring voxels will be exchangeable when it comes to predict the output class, which will lead to unreliable importance scores as derived from ML methods. To circumvent this problem, Schrouff et al. (2013) proposed to average absolute SVM weights in each region defined in a pre-existing anatomical brain atlas. This procedure improves interpretability by providing a ranking of brain regions, instead of individual voxels, according to their contribution to the prediction. In (Schrouff et al., 2018), the same authors propose to address the problem directly at the training stage with a Multiple Kernel Learning (MKL) approach. A kernel is built on each brain region defined by an atlas. Weights are then attributed to each region during the learning process, with the weights penalised using a L1-norm to enforce their sparsity. Several works have also proposed adaptations of sparse linear methods to take into account data structure. For example, Michel et al. (2010) proposed a hierarchical agglomerative clustering procedure using variance minimisation and connectivity constraints that is combined in (Jenatton et al., 2012) with a sparse hierarchical regularisation approach to fit linear models. In this approach, there are as many groups of features as there are nodes in the hierarchical tree and each group is composed of all the descendants of a node. Weights are then attributed to each group such that if one node is unselected, all its descendants will have a zero weight too.

Following these latter works with linear methods, we would like in this paper to investigate the benefit of group-based, instead of voxel-based, analyses in the context of Random Forests applied on neuroimaging data. Our first main contribution is the adaptation of Random Forests variable importance scores to rank and select groups of variables in the context of neuroimaging data. Assuming a prior division of the voxels into non overlapping groups, corresponding to different brain regions, we first propose several aggregation procedures to derive group importances from individual voxel importances. We then adapt the best permutation tests identified in Huynh-Thu et al. (2012) to turn the resulting group importances into more statistically interpretable scores. Experiments are carried out on artificial datasets to analyse the behaviour of these methods in a setting where relevant groups are perfectly known. Our second contribution is the application of these methods on our own dataset of 45 patients for the prognosis of Alzheimer's disease. We report on this dataset the main groups identified with our methods and discuss their relevance with respect to prior knowledge about the disease. The methods are applied either on groups derived from existing brain atlases from the literature or on groups identified in a data-driven manner using clustering techniques. In addition, we also study on this dataset the influence of the main Random Forests parameters on both predictive performance and stability of group importance scores, from which we derive general guidelines for practitioners.

## 2. Methods

In this paper, we are targeting the selection of relevant regions of interest in the brain for the prognosis of Alzheimer's disease with Random Forests. We assume a supervised learning setting, where we have a learning sample *LS* = (*X, Y*) composed of *n* brain images of *p* voxel intensities each collected in a matrix *X* ∈ ℝ^*n* × *p*^ and of the *n* corresponding prognosis collected in a binary vector *Y* ∈ {0, 1}^*n*^ (e.g., with 0 coding for stable MCI and 1 coding for MCI future converter). Following common machine learning terminology, voxel intensities will be also referred to as the *features* in what follows. From the learning sample, the goal is both to train a classification model that would classify as well as possible future brain images and to highlight the brain regions that are the most associated with the prognosis.

We first describe the Random Forests algorithm and how to derive variable importance scores from such models. We then describe and motivate the three aggregation functions that will be evaluated later for computing importances of groups of features and explain how these groups can be obtained. Finally, we propose adaptations at the group level of the best techniques highlighted in Huynh-Thu et al. (2012) to turn group importance scores into more statistically interpretable measures.

### 2.1. Random forests and single variable importances

Random Forests (Breiman, 2001) is a supervised learning method that builds an ensemble of *T* decision trees (Breiman et al., 1984). When inputs are numerical, a decision tree is a (typically binary) tree where each interior node is labelled with a binary test that compares one of the inputs (i.e., the intensity of a voxel) with a threshold value and where each leaf node is labelled with a prediction of the output class (0 or 1 in classification). A prediction is obtained from an ensemble of decision trees by propagating the example to test in each tree and then aggregating the predictions at the leaves reached by the example in all trees by a majority vote. In standard Random Forests, each decision tree in the ensemble is built from a bootstrap sample from the original learning sample using the standard top-down tree growing algorithm (Breiman et al., 1984) with the only difference that the best feature to split a node is searched by looking at only *K* features randomly selected among all features (with *K* ∈ {1, 2, …, *p*}).

Several methods have been proposed to derive feature importance scores from a forest. In this work, we use the mean decrease of impurity (MDI) importance with the impurity measured with Gini impurity (Breiman, 2001; Louppe et al., 2013). More precisely, for a given tree T, the importance score $I\left(x_{i},T\right)$ of a feature *x*_*i*_ is defined as:

(1) $$\begin{array}{r} I\left(x_{i},T\right)\;=\;\underset{N\in T|v\left(N\right)\;=\;x_{i}}{\sum }\frac{n\left(N\right)}{n}\Delta I\left(N\right), \end{array}$$

where the sum is over all interior nodes N in T, v(N) denotes the feature tested at node N, *n* is the size of the learning sample used to learn T, and n(N) is the number of examples reaching node N. ΔI(N) is the impurity reduction at node N defined as:

(2) $$\begin{array}{r} \Delta I\left(N\right)\;=\;I\left(N\right)-\frac{n\left(N_{l}\right)}{n\left(N\right)}I\left(N_{l}\right)-\frac{n\left(N_{r}\right)}{n\left(N\right)}I\left(N_{r}\right), \end{array}$$

where *I*(.) is the impurity function and $N_{l}$ and $N_{r}$ are respectively the left and right children of N in T. For a binary output, the Gini impurity function *I*(.) is defined by:

(3) $$\begin{array}{r} I\left(N\right)=\overset{1}{\underset{j\;=\;0}{\sum }}p_{j}\left(1-p_{j}\right)\;=\;1-p_{0}^{2}-p_{1}^{2}, \end{array}$$

where *p*_*j*_ is the proportion of examples in N that are of class *j* (with *j* ∈ {0, 1}). Finally, the importance score $I\left(x_{i}\right)$ of *x*_*i*_ in the forest is the average of its importance over the *T* trees in the forests:

(4) $$\begin{array}{r} I\left(x_{i}\right)\;=\;\frac{1}{T}\overset{T}{\underset{k\;=\;1}{\sum }}I\left(x_{i},T_{k}\right). \end{array}$$

Intuitively, a feature will get a high importance score if it appears frequently in the forest and at top nodes (leading to large $\frac{n\left(N\right)}{n}$ ratios) and if it strongly reduces impurity at the nodes where it appears.

Breiman (2001) proposed an alternative measure that computes for each feature the mean decrease of accuracy (MDA) of the forest when the values of this feature are randomly permuted in the out-of-bag samples. Both measures are mostly equivalent in practice. Experimental studies (Strobl et al., 2007) have shown that the MDI measure is biased towards features with a large number of values but this bias is irrelevant in our setting where all features are numerical. The MDI measure furthermore benefits from interesting theoretical properties in asymptotic conditions (Louppe et al., 2013) and is usually faster to compute as it does not require to perform random permutations.

### 2.2. Group importances

Importance scores as computed in the previous section will give a ranking of the hundreds of thousands of voxels that typically compose neuroimaging data. Interpreting such ranking is not easy and typically requires to map these voxels on brain maps to visually identify brain regions with a significant number of high importance voxels. Statistically, one can also expect importances at the level of voxels to be rather unreliable given the typically very small size of neuro-imaging datasets. We propose here to exploit voxel individual importances to associate instead importances to sets of voxels. To this end, and to remain as general as possible, we assume the prior knowledge of a partition of the full set of voxels into several disjoint sets, which we are interested in relating to the disease status of the patients. Ways to define such partition will be discussed in the next section. Following the terminology used in sparse linear models, we will refer to the sets of voxels in a partition as *groups*. Given individual voxel importances as computed by a Random Forests model, group importances can be derived in several ways. Denoting by *X*_*G*_ = {*x*_*i*_1__, *x*_*i*_2__, …, *x*_*i*_*#G*__} the set of features in a given group *X*_*G*_ of *#G* voxels, we will investigate three aggregation functions to derive group importances, computing respectively the sum, the average, and the max of the importances of the features in the group:

$$\begin{array}{l} I_{\text{sum}}\left(X_{G}\right)=\overset{\#G}{\underset{j\;=\;1}{\sum }}I\left(x_{i_{j}}\right),\;I_{\text{avg}}\left(X_{G}\right)=\frac{1}{\#G}\overset{\#G}{\underset{j\;=\;1}{\sum }}I\left(x_{i_{j}}\right),\\ I_{\text{max}}\left(X_{G}\right)=\underset{j\;=\;1,\ldots ,\#G}{\max}I\left(x_{i_{j}}\right). \end{array}$$

Louppe et al. (2013) have shown that the sum of the MDI importances of all features represents the total amount of class impurity reduction brought by the forest. Taking the sum of the importances is thus the most natural choice: the importance of a group is the total class impurity reduction brought by the features from the group. The sum has however the drawback that it is potentially biased towards groups of larger sizes. Indeed, large groups have more chance to have their features selected when building the forest. The average avoids any bias due to differences in group cardinality but has the drawback that a group can not be important if only a small proportion of its features are important. Finally, taking the maximum of the importances in the group assumes that the feature of highest importance alone is representative of the group importance. In other words, it considers that a group is important as soon as one of its feature is important. As it is unclear a priori which aggregation function would work best in practice, we will compare all of them on both the artificial and real datasets.

### 2.3. Group definition

Computing group importances requires the availability of a partition of the voxels into groups. In this work, we will only consider partitions into contiguous sets of voxels, with groups thus corresponding to non-overlapping brain regions. Such partition will be referred to as an *atlas*. Two kinds of atlases can be investigated: (1) atlases derived manually from prior knowledge of the brain structure, such as the automated anatomical labelling (AAL) atlas (Tzourio-Mazoyer et al., 2002), and (2) data-driven atlases derived automatically from the learning sample using clustering techniques (e.g., Thirion et al., 2014). We will focus our analysis in the rest of the paper on the first family of atlases, which leads to more interpretable results. Some experiments with data-driven atlases on the real dataset are nevertheless presented in the Supplementary Materials.

### 2.4. Group selection methods

Typically, most groups will receive a non-zero importance from the Random Forests model. From an importance ranking, it is therefore difficult to distinguish the truly relevant groups from the irrelevant ones. In this section, we propose to adapt at the group level, several methods that have been proposed in the literature to transform ML based importance scores into more statistically interpretable measures similar to *p*-values. This will help determining a threshold in the ranking below which all groups can be considered as irrelevant.

Beyond an improvement of interpretability, applying these techniques to groups of features instead of individual features has several additional advantages. First, some of these methods are very computationally demanding, as they require for each score computation, and thus for each feature, to retrain Random Forests several times with randomly permuted features or labels. This makes the application of the most demanding methods impossible at the level of voxels. Working at the group level, on the other hand, will reduce the number of scores to evaluate to a few hundreds only (depending on the size of the atlas) and therefore will strongly reduce computing times. Second, from a statistical point of view, one can expect aggregated group scores to be more stable than individual voxel scores. Combined with the strong reduction of the number of considered features, we expect that working at the group level will thus also improve the statistical power of the tests, which will lead to the selection of more significant brain regions than when dealing directly with voxels.

Huynh-Thu et al. (2012) have carried out an empirical comparison of several techniques to turn ML scores into statistical scores in the context of bioinformatics studies. We will present below the adaptation for groups of the three best methods identified in this study. Two of these methods, the *conditional error rate* (CER) and the *estimated false discovery rate* (eFDR), are based on models retrained on randomly permuted version of the original features, and one method, *mProbes*, train models with additional random features (called probes). mProbes and CER controls the family wise error rate and are recommended by Huynh-Thu et al. (2012) when a very low false positive rate is targeted (i.e., to minimize the number of groups selected that are not truly relevant), while the eFDR is comparatively less conservative as it controls the FDR.

In our presentation of these methods, we assume that, from the learning sample *LS*, our machine learning algorithm has provided a score of importance *s*_*i*_ for each group, with *i* = 1, …, *G*, using any aggregation function. Without loss of generality, groups are assumed to be ordered according to their importance score, such that *g*_*i*_ is the *i*th group in this ranking.

#### 2.4.1. Multiple testing with random permutations

The goal of the CER and eFDR methods is to control the “family-wise error rate” (FWER) and the “false discovery rate” (FDR) respectively when choosing a threshold on the group importance scores. The FWER is the probability of selecting one or more false positives (irrelevant groups) among the groups that are identified as relevant, while the FDR is the expected rate of false positives among them (Storey and Tibshirani, 2003).

The *conditional error rate* method has been introduced by Huynh-Thu et al. (2008) to overcome the limitations of the classic permutation-based FDR estimation techniques used for univariate statistical tests (Ge et al., 2003). When applied to multivariate importance scores, these methods indeed usually overestimate the FDR, which leads to unreliable selections (Huynh-Thu et al., 2008). The CER wants to estimate the probability to include an irrelevant group when selecting all groups until group *g*_*i*_ in the ranking. For group *g*_*i*_, the conditional error rate is defined by:

(5) $$\begin{array}{r} CER_{i}=P\left(\underset{k\;=\;i,\ldots ,G}{\max}s_{k}^{p}\geq s_{i}|H_{R}^{1\to i-1},H_{I}^{i\to G}\right), \end{array}$$

where $H_{R}^{1\to i-1}$ is the hypothesis that groups *g*_1_ to *g*_*i* − 1_ are relevant, $H_{I}^{i\to G}$ is the hypothesis that group *g*_*i*_ and all the groups ranked below *g*_*i*_ are irrelevant and $s_{k}^{p}$ is the importance score of the group k under these assumptions. The *CER*_*i*_ score for a given group *g*_*i*_ is estimated by retraining Random Forests on randomly permuted data (with *P* repetitions): class labels and features in groups *g*_1_ to *g*_*i* − 1_ are kept unchanged to simulate $H_{R}^{1\to i-1}$, while features in groups *g*_*i*_ to *g*_*G*_ are randomly permuted to simulate $H_{I}^{i\to G}$ (using the same permutation vector for all features so as to remain as close as possible to the original data distribution). The number of relevant groups is then computed as the maximum rank *r* for which *CER*_*r*_ is lower than a pre-defined risk α (with α typically set to 0.05).

In our previous work (Wehenkel et al., 2017), we proposed the following adaptation of the conditional error rate:

(6) $$\begin{array}{r} CER_{i}^{r}=P\left(rank\left(g_{i}\right)\leq i|H_{R}^{1\to i-1},H_{I}^{i\to G}\right), \end{array}$$

where the relevance score is replaced by the rank. The idea behind this score is that a group which is really relevant should not be as well or better ranked than it is in the original data once we break the link between the features in this group (and in all groups that follow in the original order) and the output through the randomisation procedure. This adaptation is expected to be less restrictive than the CER in (5) and thus using the same α threshold, it should lead to a higher true positive rate at the expense however of the false positive rate.

Ge et al. (2008) propose to estimate the FDR with

(7) $$\begin{array}{r} eFDR_{i}=E\left[\frac{V_{i}}{V_{i}+i-1}|H_{R}^{1\to i-1},H_{I}^{i\to G}\right], \end{array}$$

where $H_{R}^{1\to i-1}$ and $H_{I}^{i\to G}$ are the same hypotheses as in (5) and *V*_*i*_ is the number of false positives. *eFDR*_*i*_ is estimated in the following way. $H_{R}^{1\to i-1}$ and $H_{I}^{i\to G}$ are simulated using the same group-based permutation procedure as for the CER. *V*_*i*_ is computed, for each permutation, as:

(8) $$\begin{array}{r} V_{i}=\underset{k\;=\;1,\ldots ,G-i+1}{\max}\left\{k:s_{\left(1\right)}^{p}\geq s_{i},s_{\left(2\right)}^{p}\geq s_{i+1},\ldots ,s_{\left(k\right)}^{p}\geq s_{i+k-1}\right\}, \end{array}$$

with $s_{\left(k\right)}^{p}$ the *k*th largest value in $\left\{s_{i}^{p},\ldots ,s_{G}^{p}\right\}$ and $s_{k}^{p}$ the relevance score of group *g*_*k*_ calculated from the randomly permuted data. *V*_*i*_ is thus the maximal number of randomly permuted groups, ordered according to their importance, whose importance exceeds the importance of the matching group ordered according to the original importance scores.

#### 2.4.2. Utilisation of random probes

A third method suggested by Huynh-Thu et al. (2012) is the mProbes approach, which is a variant of a method proposed in Tuv et al. (2009). When applied at the feature level, the idea of this method is to introduce as many random features as the input matrix contains originally, where each new random feature is generated by randomly permuting the values of one original feature. A Random Forests model is trained on the resulting dataset and is used to rank the features according to their importance. The experiment is repeated *P* times with new permutations and the FWER for a given original feature is estimated by the proportion of the *P* runs where at least one random feature is better ranked than this feature.

The procedure can be easily adapted to groups. A random group is obtained from each original group by randomly shuffling the features within the group. Features within a group are permuted using the same permutation vector to keep feature correlations unchanged inside the group. The FWER for a group *g*_*i*_ in the original ranking is then estimated by the proportion of Random Forests runs (among *P*) where at least one randomly permuted group is ranked better than group *g*_*i*_.

This method is more efficient than CER and eFDR since it only requires to rerun Random Forests (with twice as much features however) *P* times, compared to *G* × *P* times with CER and eFDR, to get all group statistics.

## 3. Data and assessment protocol

### 3.1. Artificial datasets

In order to validate our methods in a situation where truly relevant features are already known, we generate artificial datasets for a linear classification problem. Artificial datasets construction is inspired from the linear datasets construction used in (Huynh-Thu et al., 2012).

Each dataset contains *p* features denoted (*x*_1_, …, *x*_*p*_) that are divided a priori into *g* groups denoted (*G*_1_, *G*_2_, …, *G*_*g*_), with the size of group *G*_*i*_ denoted ♯*G*_*i*_ (We used *p* = 500 and *g* = 50 in all our experiments). Without loss of generality, we assume that features are ordered following the group distribution such that group *G*_*i*_ is composed of features $x_{\left(\overset{i-1}{\underset{k=1}{\sum }}♯G_{k}\right)+1}$ to $x_{\overset{i}{\underset{k=1}{\sum }}♯G_{k}}$, ∀*i* = 1, …, *g*. To generate group of random sizes, we proceed as follows. We draw *g* − 1 cut-off values at random without replacement from {1, …, *p*}. Denoting by (*c*_1_, …, *c*_*g* − 1_) these values in increasing order and defining *c*_0_ = 0 and *c*_*g*_ = *p*, the size of the *i*th group (*i* = 1, …, *g*) is then set to *c*_*i*_ − *c*_*i* − 1_.

Among these groups, *R* are relevant and *I* = *g* − *R* are irrelevant by construction. Let us denote by *G*^*R*^ and *G*^*I*^ respectively the sets of relevant and irrelevant groups. Values of the features in the irrelevant groups are drawn independently of each other from a normal distribution, ie., $x_{i}\sim N\left(0,1\right),\forall x_{i}\in g\text{ and}\forall g\in G^{I}$. For each relevant group $G_{k}\in G^{R}$, one feature $x_{k}^{R}$ is first drawn from a normal distribution such that $x_{k}^{R}\sim N\left(0,1\right)$ for *k* = 1, …, *R*. The output *y* is then computed from the $x_{k}^{R}$ features as follows:

(9) $$\begin{array}{r} y=sgn\left(\overset{R}{\underset{k=1}{\sum }}w_{k}x_{k}^{R}\right), \end{array}$$

where the values of the coefficients *w*_*k*_ are drawn uniformly in [0, 1]. Features $x_{k}^{R}$ are not put directly in the dataset. Instead, features within each relevant group are generated each as a noisy copy of $x_{k}^{R}$, obtained by adding a normal N(0,1) noise to $x_{k}^{R}$. The motivation for this procedure is to create a non perfect correlation between the features within the relevant group, so that they are jointly more informative about the output than individually. Finally, 1% of the output values have been randomly flipped to make the problem harder to solve.

### 3.2. Real dataset

Forty-five patients presenting MCI were enrolled in a longitudinal study achieved by the Cyclotron Research Centre in University of Liège, Belgium. More precisely, patients were selected based on Petersen's criteria (Petersen and Negash, 2008) for MCI, including memory complaints, objective memory deficits on neuropsychological testing, no evidence of global cognitive decline and preserved activities of daily living. At the beginning of the study, one Fludeoxyglucose (^18^F-FDG) positron emission tomography (PET) image was recorded for each patient. During the next fours years, patients were followed and evaluated repeatedly with neuropsychological tests. Conversion was detected as soon as a patient fulfilled the diagnosis criteria for Alzheimer's disease at a follow-up assessment, that is, objective deficit in more than two cognitive domains, general cognitive decline and significant reduction of autonomy in everyday life activities. Along the time of the study, several individuals converted from MCI to Alzheimer's disease and, at the end of the study, the total number of converters (MCIc) was 22. Demographic details about patients at their entrance in the study are reported in Table S1. It is worth noting that data labels have been somehow artificially binarised in two classes, MCIc and stable MCI. Indeed after the four years of follow up, some MCI patients could potentially still develop the disease. The real problem consists in distinguishing the patients who will develop the disease in the next four years and those who will not, as well as identifying the relevant regions for this prediction.

As required, the protocol of the study was accepted by University Ethics Committee in Liège. All patients received a written and oral description of the study and then provided a written consent. Concerning the acquisition of the images, they were performed 30 min after injection of the ^18^F-FDG radiopharmaceutical, by means of a Siemens ECAT HR+ PET gamma camera (3D mode; 63 image planes; 15.2cm axial field of view; 5.6 mm transaxial resolution and 2.4 mm slice interval). Images were reconstructed using filtered backprojection including correction for measured attenuation and scatter using standard software.

After acquisition, images were pre-processed using SPM8. Since no structural MRI was available, all PET images were spatially normalised to the MNI reference space using the template matching approach implemented in SPM8 (Ashburner et al., 1999; Penny et al., 2011), assuming that the signal decrease in the hypometabolic area(s) was not significantly affecting the spatial transformation. Spatial normalisation was followed by an intensity scaling by cerebellar uptake as the cerebellum is assumed to be unaffected by the disease (Dukart et al., 2010). The cerebellum was delineated according to the automated anatomical labelling (AAL) atlas (Tzourio-Mazoyer et al., 2002). To finally obtain a feature vector for each patient, a mask was applied to extract only the voxels included inside the brain volume. This stage gave rise to a feature vector composed of a little bit less than 220,000 variables per image.

### 3.3. Atlas-based parcelling

For artificial datasets, the group structure is perfectly known in advance and it was used to define voxel groups. For real datasets, brain atlases are in general available for the sake of result interpretation. We thus decide to evaluate our methods with a prior division of the brain according to the brain structure as it is the simplest choice and the most interpretable one. In particular, the atlas we use is the AAL atlas (Tzourio-Mazoyer et al., 2002), composed of 116 distinct anatomical regions. The AAL atlas provides neuroanatomical labels only for gray matter areas. Our approach is thus by default limited to the gray matter. In addition, we provide in the Supplementary Materials results obtained with several data-driven atlases.

### 3.4. Group selection

Group importance scores are generated by Random Forests of 1,000 trees by default, but larger values are also explored. Regarding the number of features randomly drawn at each split, i.e., the parameter *K*, we mainly explore two settings: *K* = 1 and $K=\sqrt{p}$. $K=\sqrt{p}$ is a common default setting which usually leads to good predictive performance on classification problems (Geurts, 2001). *K* = 1 is an extreme setting, which amounts at selecting the feature for splitting a node fully at random. While this value of *K* is not expected to lead to optimal predictive performance, we tested this value for two reasons. First, it makes the tree construction very fast and independent of the total number of features. Second, it was shown in the theoretical analysis of Louppe et al. (2013) to be the only setting that guarantees a fair treatment of all features by avoiding any masking effects between them. Indeed, when two features convey about the same information about the output, using a value of *K* > 1 might prevent one of them to be selected at a given node when it is in competition with the other one. As a consequence, the importance of one of the two features will be greater than the importance of the other, while both features are almost equally important. Note however that using *K* = 1 is likely to lead to importance estimates of higher variance than using $K=\sqrt{p}$ and therefore to require building more trees for these estimates to reach convergence.

As in (Huynh-Thu et al., 2012), the permutation scheme for all statistical measures considers *P* = 1, 000 repetitions and the α threshold on all statistical scores is fixed to 0.05.

### 3.5. Performance metrics

Each method gives rise to a subset of relevant groups. In the case of artificial data, we are directly able to verify if this subset truly contains the relevant groups. Method performance is thus evaluated in the case of artificial problems with the precision $\frac{TP}{S}$ and recall $\frac{TP}{P}$ with *TP* the number of truly relevant groups that have been selected, *S* the total number of selected groups and *P* the total number of truly relevant groups in the problem.

Independently of the use of a group selection method, it is interesting also to evaluate the quality of the group importance ranking. This ranking can be evaluated by computing the area under the precision-recall curve (AUPR), which plots the evolution of precision vs. recall when selecting an increasing number of groups at the top of the ranking. The AUPR is equal to 1 when all truly relevant groups appear at the top of ranking and it is close to *R*/*g*, with *g* the number of groups, when groups are ranked randomly. To provide further comparison, we also evaluate the highest precision that can be achieved for a unitary recall and the highest recall that can be achieved for a unitary precision, respectively denoted *rec-1* and *prec-1* in the Results section. *rec-1* corresponds to the most conservative selection method that wants to avoid any false positive and *prec-1* corresponds to a method that does not want to miss any truly relevant feature. Note that these two methods are purely theoretical methods that can not be implemented in practice without a perfect knowledge of the relevant groups. Their performance is provided as baselines for comparison.

For the real dataset, as the truly relevant features (voxels or regions) are unknown, we can not evaluate performances through precision and recall as on the artificial datasets. As commonly done, we thus evaluate selection methods by comparing the regions found with the regions identified in the Alzheimer's disease literature. In addition, we also evaluate the different aggregation functions through the classification errors (estimated by cross-validation) of models trained using the most relevant groups found by each function. Finally, we further compare our methods with the MKL approach proposed in (Schrouff et al., 2018) using the AAL atlas. This method is close to ours in that it also performs feature selection at the level of regions. The *C* hyper-parameters of this method is tuned using an internal ten-fold cross-validation loop (with *C* optimised in 10^[−3:1:3]^).

## 4. Results

We analyse in this section results obtained with artificial and real datasets.

### 4.1. Artificial datasets

Our goal in this section is to highlight the main properties of the group selection methods in a setting where relevant groups are known and one can thus assess quantitatively the capacity of the methods at selecting the correct groups.

#### 4.1.1. Comparison of the aggregation functions

**Figure 1:**
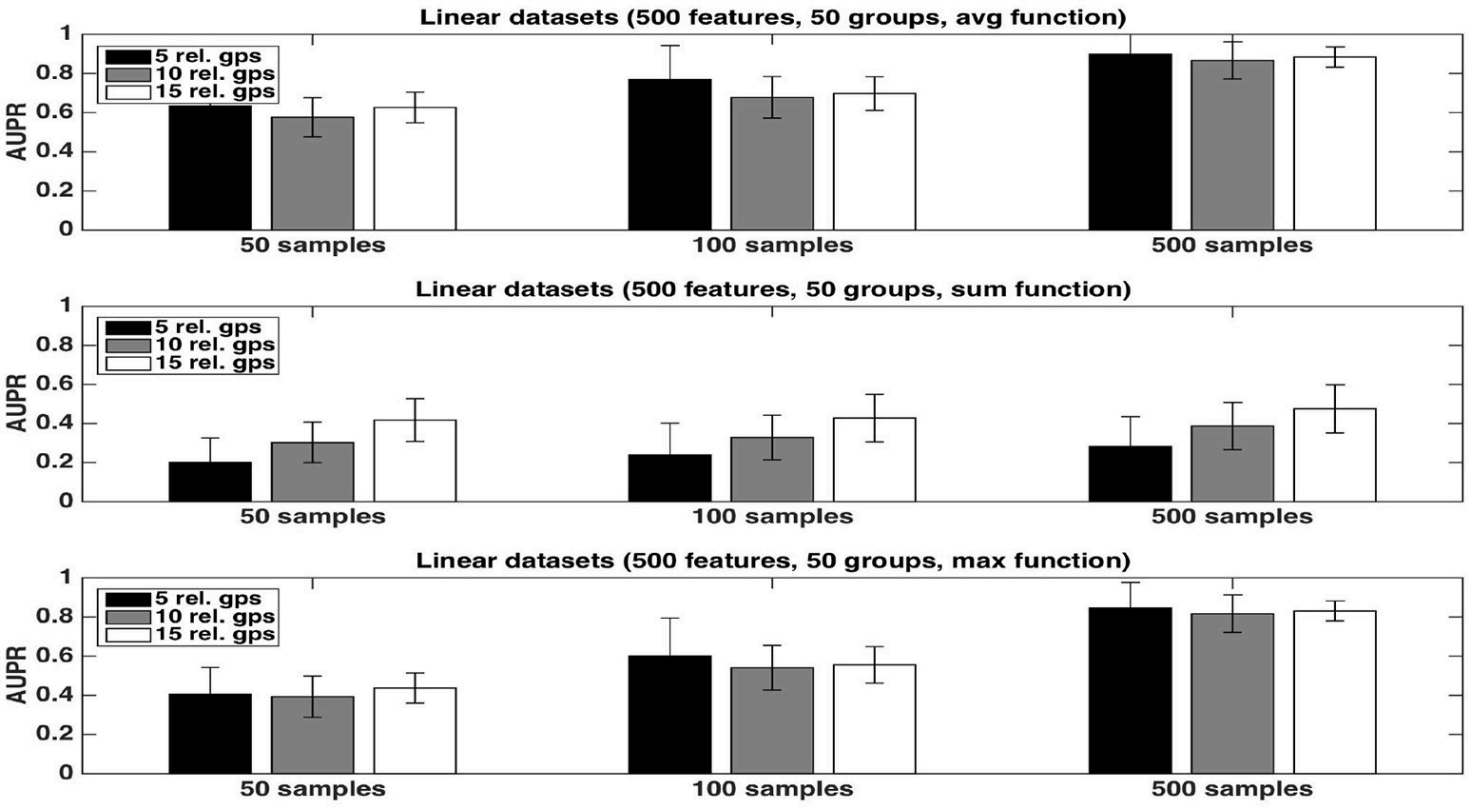
Artificial datasets. AUPRs of Random Forests (T=1,000 and *K* = 1) ranking method with different aggregation functions, for different numbers of relevant groups and different sample sizes. Top is on *average* function, middle on *sum* function and bottom on *max* function. The AUPR values were averaged over 20 datasets in each case.

**Figure 2:**
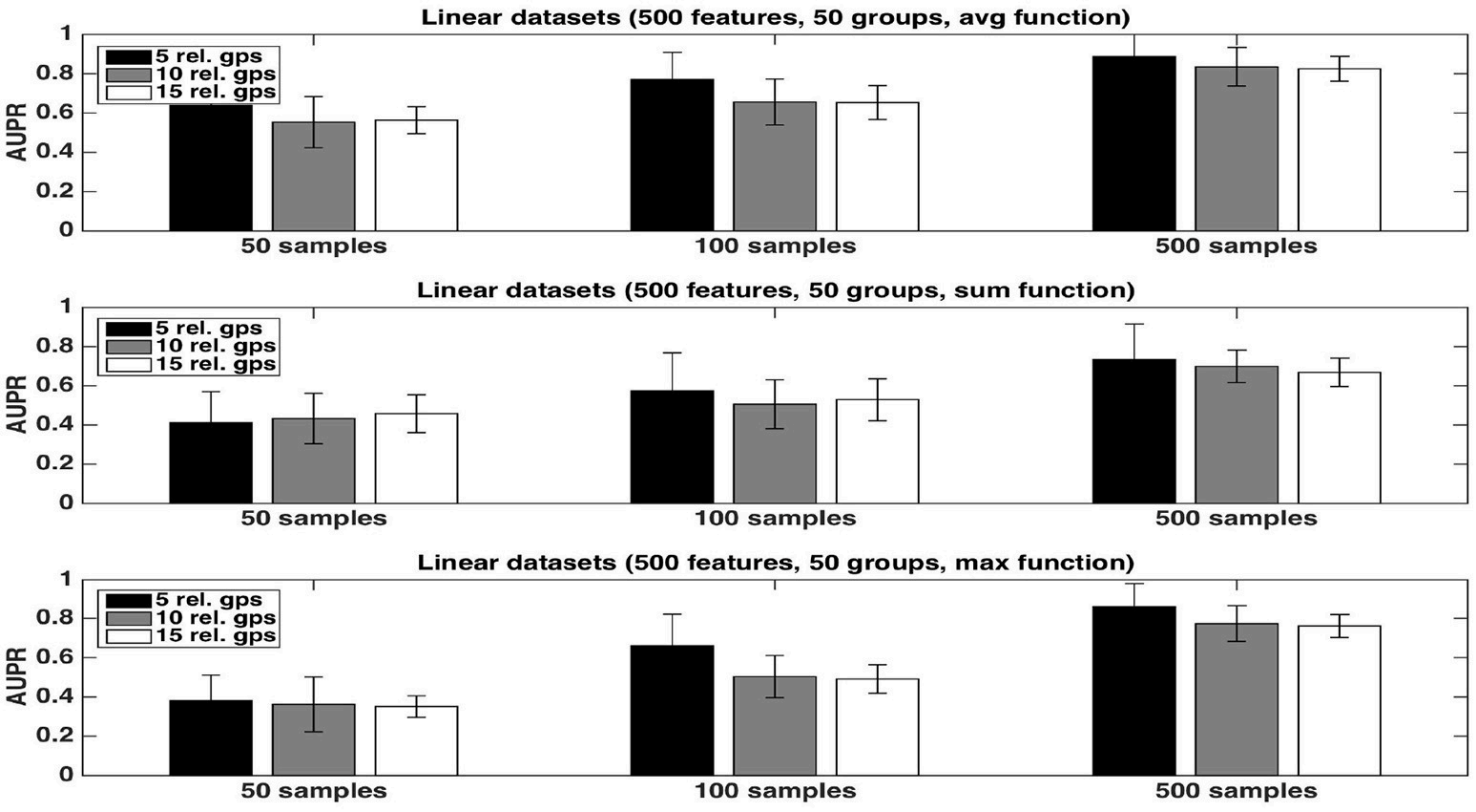
Artificial datasets. AUPRs of Random Forests (T=1,000 and $K=\sqrt{p}$) ranking method with different aggregation functions, for different numbers of relevant groups and different sample sizes. Top is on *average* function, middle on *sum* function and bottom on *max* function. The AUPR values were averaged over 20 datasets in each case.

We first evaluate the quality of the group rankings obtained with the three aggregation functions: the *average*, the *sum*, and the *maximum*. AUPRs with the three functions are shown in Figures 1, 2, respectively with *K* = 1 and $K=\sqrt{p}$, in both cases for an increasing number *R* of relevant groups and an increasing number of samples. All results are averaged over 20 randomly generated datasets.

The *average* function is clearly producing the best rankings in all settings. The *max* function is competitive in large sample settings but it is clearly inferior with the smallest sample size. The *sum* is inferior to the two other functions in all settings, but its AUPRs are especially very bad when *K* = 1. We attribute the bad performance of the sum in this setting to its bias towards groups of large size. Indeed, when *K* = 1, features used to split are selected uniformly at random among all features and thus there are more splits based on features from larger groups in the trees. As a consequence, even if each feature of a large irrelevant group will receive a low importance, when summing them, the importances of their group might still be comparable with the importances of small relevant groups. As a confirmation of this effect, we indeed observe a strong correlation between group importances and group sizes when using the sum function. Although still present, the effect is reduced with $K=\sqrt{p}$, as in this case, features from irrelevant groups are put in competition with features from relevant groups and have thus less chance to be selected in the trees.

As expected, the AUPRs increase in all cases when the number of samples increases. Except for the *max* function, the AUPRs slightly decrease with the number of relevant groups.

#### 4.1.2. Comparison of statistical scores

**Figure 3:**
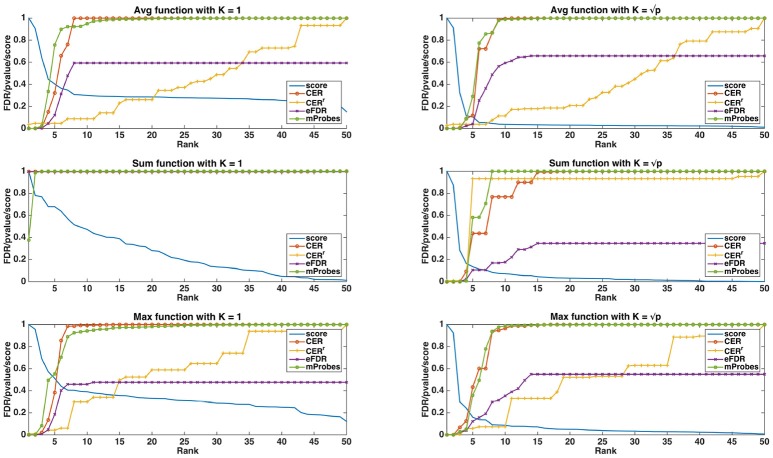
Artificial datasets (500 features, 50 groups, 5 relevant groups, 100 samples). Curves of the importance scores (T=1,000, *K* = 1 and $K=\sqrt{p}$) and the different selection methods obtained on a linear dataset. The curve labelled as “Score” is the group importance score.

In Figure 3, we show, both for *K* = 1 and $K=\sqrt{p}$, how the different statistical group measures evolve with the rank for the three aggregation functions. In all cases, the group importances decrease rapidly and then much more slowly, suggesting that only a few groups contain most of the information. The only exception is the maximum group importance with *K* = 1, which decreases slowly from the beginning. Statistical scores mostly show the expected behaviours. CER and mProbes, which both estimate the FWER, have similar evolutions. The statistical measures they compute remain close to zero for 3 or 4 groups and then increase very abruptly towards 1. As expected, eFDR, which estimates the FDR, leads to a slower increase of its statistical score towards 1 also after 3 or 4 groups. CER^*r*^ has the slowest progression in all cases, except with the *sum* function and $K=\sqrt{p}$ where it increases more rapidly than the other scores. All statistical scores are directly close to 1 with the *sum* function when *K* = 1, showing that the ranking provided by this group importance does not behave well. Note that the point where most statistical scores start raising is consistent with the position in the ranking at which irrelevant groups starts appearing: with the *average*, the first irrelevant group is at the fifth position in the ranking, whatever *K*. With the *sum*, the fourth group is the first irrelevant one for both *K*. With the *max*, the first irrelevant group is the first one with *K* = 1 and the fifth one with $K\;=\;\sqrt{p}$.

Table 1 compares methods when they are used for feature or group selection directly. We report in this table the average (over 20 datasets) number of groups selected by all four methods, the average number of features that are contained in these groups, and the average number of relevant groups among the selected ones. As a comparison, we also provide in the same table, the number of features and (relevant) groups selected when the four statistical scores are computed at the level of features instead of groups. In this case, a group is considered as selected as soon as one of its feature is selected.

**Table 1:** Average number of features selected (α = 0.05) and number of corresponding groups and relevant groups on linear artificial datasets (500 variables, 50 groups, 5 relevant groups, and 100 samples) for each method.

		**CER**			**CER**^*****r*****^			**eFDR**			**mProbes**		
		**feat**	**gps**	**rel. gps**	**feat**	**gps**	**rel. gps**	**feat**	**gps**	**rel. gps**	**feat**	**gps**	**rel. gps**
*K* = 1	RF	7.15	1.55	1.55	47.75	20.35	3.70	11.85	1.85	1.75	1.75	0.2	0.2
	*avg*	18.50	2.20	**2.20**	14.85	1.40	1.30	21.45	2.70	2.60	16.00	1.75	**1.75**
	∑	5	0.30	0.30	7.75	0.45	0.45	7.5	0.40	0.40	7.40	0.35	0.35
	max	14.90	1.60	1.60	28.35	3.10	2.55	17.45	1.75	1.65	11	1.10	1.10
$K=\sqrt{p}$	RF	7.05	1.45	1.45	61.10	23.80	3.80	11.20	2.05	1.75	11.05	1.65	1.65
	*avg*	19.80	2.75	**2.75**	25.90	2.65	2.15	22.55	3.15	3.05	20.45	2.70	**2.70**
	∑	16.35	1.40	1.40	23.55	2.20	2.15	17.35	1.55	1.55	22.75	2.00	2.00
	max	12.50	1.65	1.65	35.90	4.00	2.90	14.50	1.80	1.75	12.95	1.75	1.75

*RF means Random Forests without any aggregation function. Bold text and underlined text are for best number of relevant groups over all aggregation functions and over all selection methods respectively*.

Several interesting observations can be made from this table. When working at the group level, the *average* aggregation leads to the highest number of selected groups with CER, mProbes, and eFDR. With the CER^*r*^, more groups are found with the *max* aggregation. Except with the CER^*r*^, it is interesting to note that working at the level of features instead of groups actually leads to the selection of less groups than using the average group importance. This supports our previous argument that working at the group level is actually beneficial in terms of statistical power. The CER and the mProbes methods seem to only find relevant groups since the average number of selected groups always exactly matches the number of selected relevant groups. For the eFDR, a few selected groups are actually irrelevant as these two numbers do not exactly match. The CER^*r*^ on the other hand seems to select much more irrelevant groups. In particular, its precision is very poor when it is used at the feature level. These results will be confirmed in the next section. Finally, for all methods, using $K=\sqrt{p}$ allows to find more (relevant) groups that using *K* = 1.

#### 4.1.3. Precision and recall

**Figure 4:**
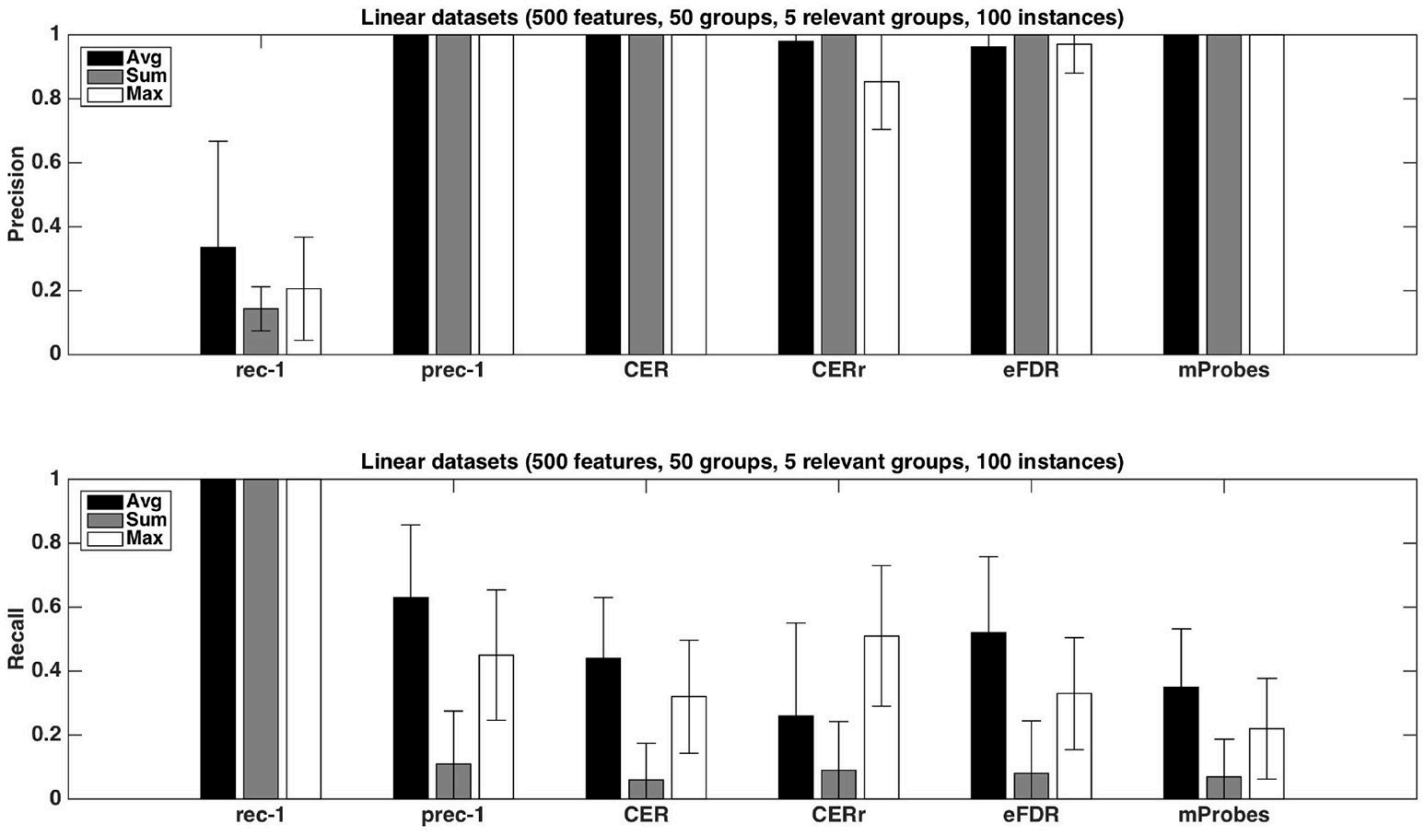
Artificial datasets. Precision and recall of each selection method (T=1,000 and *K* = 1) for the three different aggregation functions investigated. We used a selection threshold α = 0.05. The precision and recall values were averaged over 20 datasets in each case.

Figure 4 shows the precision and recall of each method with the different aggregation functions averaged over 20 datasets, with *K* = 1. As already noticed from Table 1, the precision is close to one for all methods except the CER^*r*^ with *max*. None of the proposed methods can reach a recall equal or higher than the one of prec-1. Except for CER^*r*^ for which the recall is the highest when *max* is used, the other methods obtain the best results with the *average* aggregation function. eFDR with averaging obtains the highest recall among the proposed methods, while the recalls of mProbes and CER are very close.

**Figure 5:**
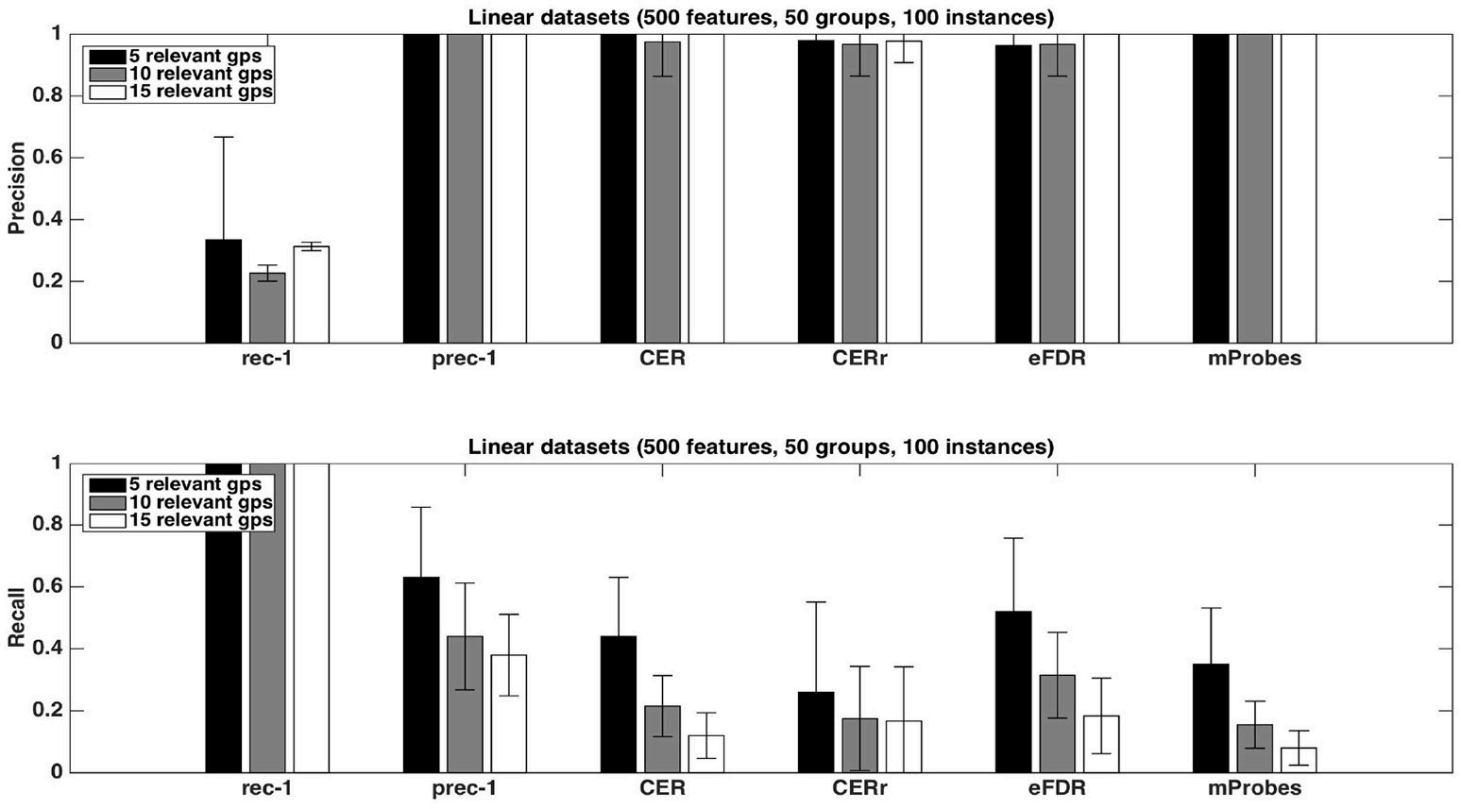
Artificial datasets. Precision and recall of each selection method (T=1,000 and *K* = 1) for different numbers of relevant groups. We used the *average* function as ranking method and a selection threshold α = 0.05. The precision and recall values were averaged over 20 datasets in each case.

Figure 5 shows the impact of the number of relevant groups on precision and recall, with the *average* function. Precisions are mostly unaffected while recalls decrease when the number of relevant groups increases. Given that the recall is the proportion of relevant groups found by the methods, this suggests that the number of selected groups does not grow proportionally with the number of relevant groups.

**Figure 6:**
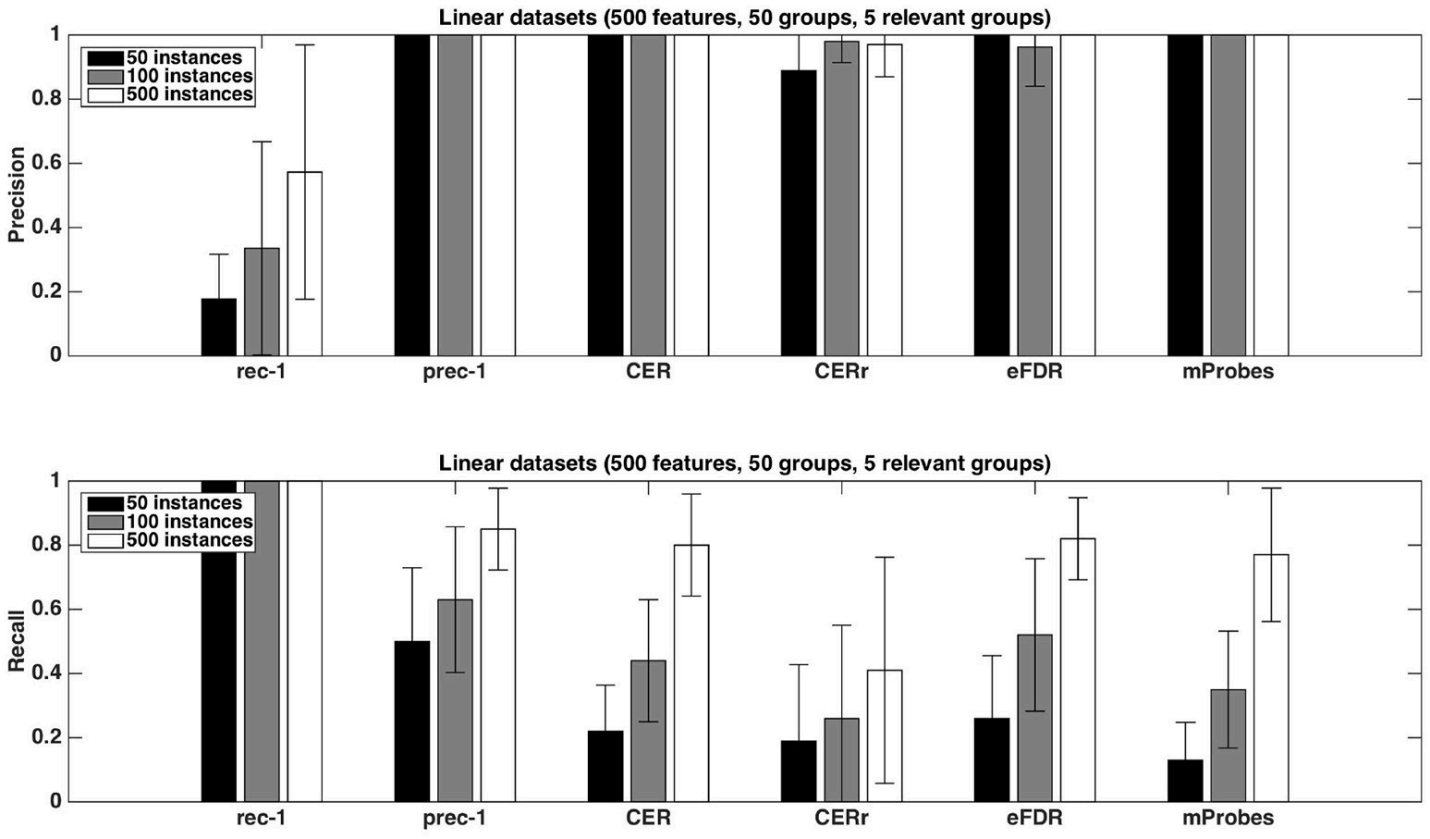
Artificial datasets. Precision and recall of each selection method (T=1,000 and *K* = 1) for different numbers of sample sizes. We used the *average* function as ranking method and a selection threshold α = 0.05. The precision and recall values were averaged over 20 datasets in each case.

Finally, as expected, increasing the number of samples in datasets helps to improve the performances. This phenomenon is illustrated in Figure 6. With 500 samples, recall of CER, eFDR and mProbes are getting closer to recall of prec-1. Unfortunately, such a ratio is in general not encountered in neuroimaging problem. Improvement of recall value is really less impressive for CER^*r*^. This latter method also exhibits a lower precision than the other ones.

#### 4.1.4. Summary

The comparison of the aggregation functions shows that the *average* and the *max* functions work better than the *sum* function, due to a bias of this latter aggregation function towards large groups, in particular when *K* = 1. The *average* function provides better AUPR scores than the *max* in small sample setting, while both methods are close with larger sample sizes. Concerning RF parameters, $K=\sqrt{p}$ is clearly a better choice than *K* = 1 as it enables to detect more relevant groups, at the expense however of computing times. Among statistical scores, CER and mProbes select no false positives while eFDR selects a few and CER^*r*^ a lot. Finally, our results show that working at the group level is beneficial because it allows to select more relevant groups than working at the level of individual features.

### 4.2. Real dataset

In this section, we present results obtained with the group selection methods on a dataset related to Alzheimer's prognosis. This dataset constitutes a very challenging problem for ML methods, as it contains a very large number of features (around 200, 000 voxels) and only few dozens of samples (45 patients). We will first study in section 4.2.1 the predictive performance of Random Forests on this dataset (in comparison with the MKL method) and study the impact of its main parameters, *T* and *K*, on both error rates and group ranking. In section 4.2.2, we will then analyse the behaviour of the group selection methods, depending on the aggregation function and Random Forests parameters. Finally, in section 4.2.3, we will analyse the groups found by these methods in the light of prior knowledge about Alzheimer's prognosis.

#### 4.2.1. Predictive performance and group ranking

**Figure 7:**
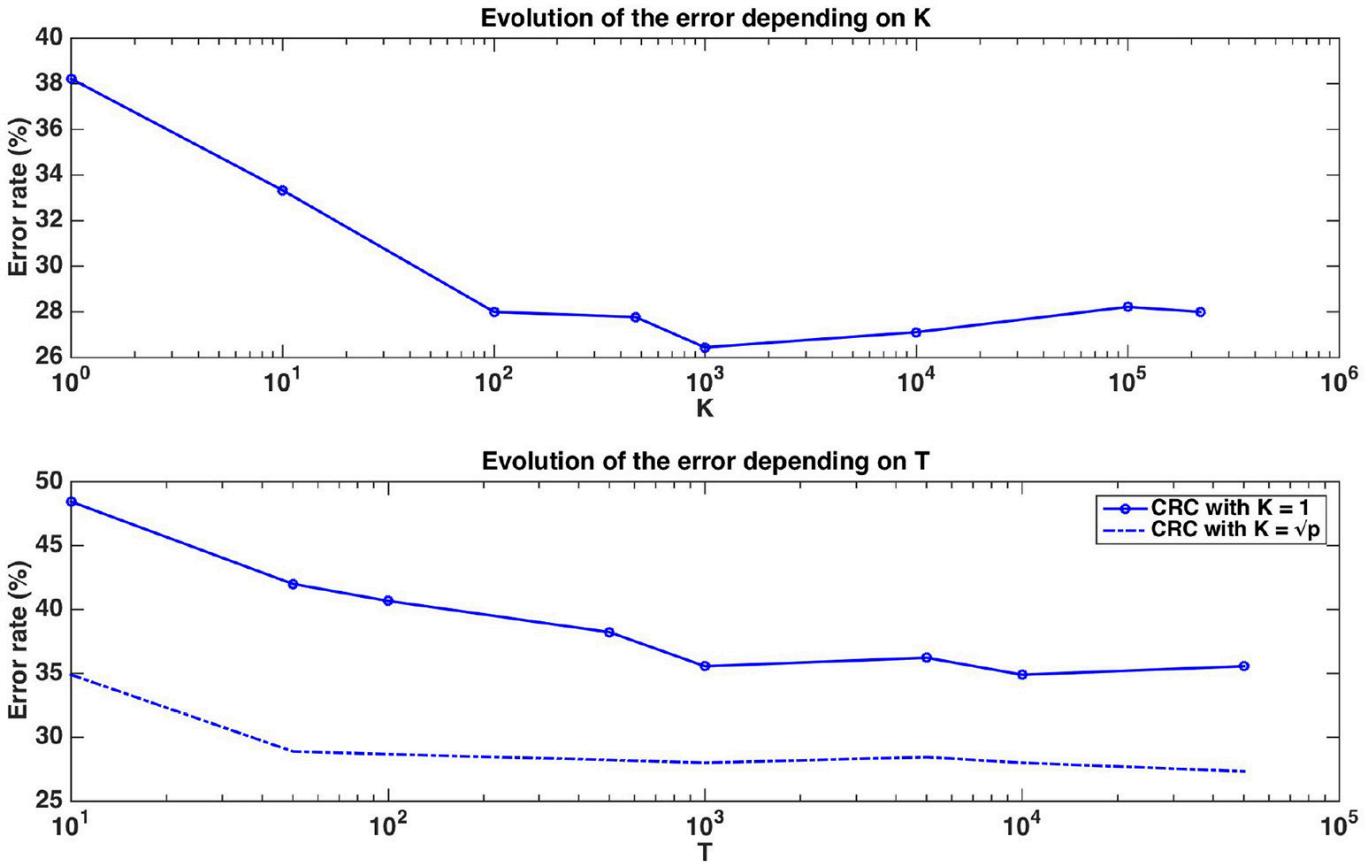
Real dataset. Error rates of a Random Forests classifier as a function of *K* parameter value for T=1,000 (top figure, x-axis in log scale) and as a function of the number of trees *T* for *K* = 1 or $K=\sqrt{p}$ (bottom figure, x-axis in log scale). Errors are evaluated with a ten repeated ten fold cross validation procedure.

Figure 7 shows the evolution of the error rate depending on parameters *K* and *T*. Errors in this figure are obtained as averaged over ten repeated ten fold cross-validation runs. The error rate for T=1,000 reaches its minimum value at around K=1,000 (which is close to $K=\sqrt{p}$). Moreover, the error decreases as the number of trees *T* composing the forest increases and stabilises at around T=1,000. With default parameters (T=1,000 and $K=\sqrt{p}$), Random Forests reach an error rate of 28.89%, which is much better than the error rate of a classifier always predicting the majority class (49%). This suggests that despite the small size of the dataset, Random Forests are able to extract meaningful information from the data.

**Figure 8:**
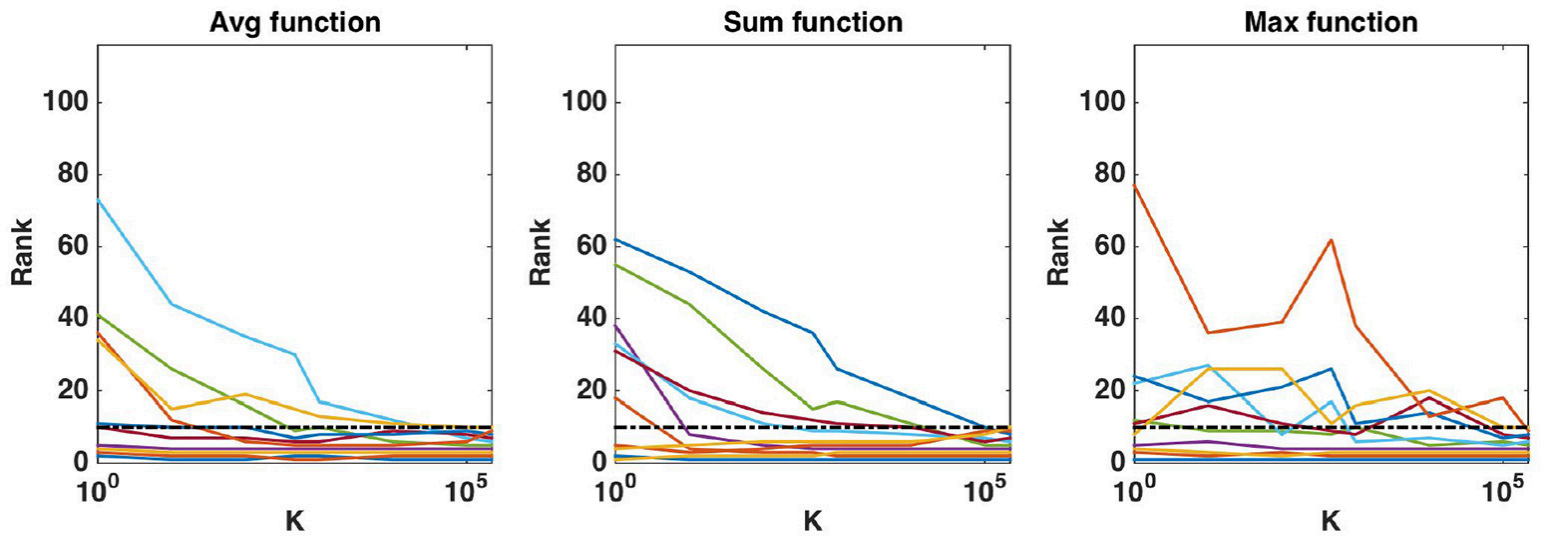
Real dataset. Evolution of the rank as a function of *K* parameter value (in log scale) for the first ten regions obtained (*T* = 10, 000 and *K* = *p*). Importance scores are computed for the AAL atlas and for each aggregation function. Black horizontal dotted line represents the 10*th* ranking position.

**Figure 9:**
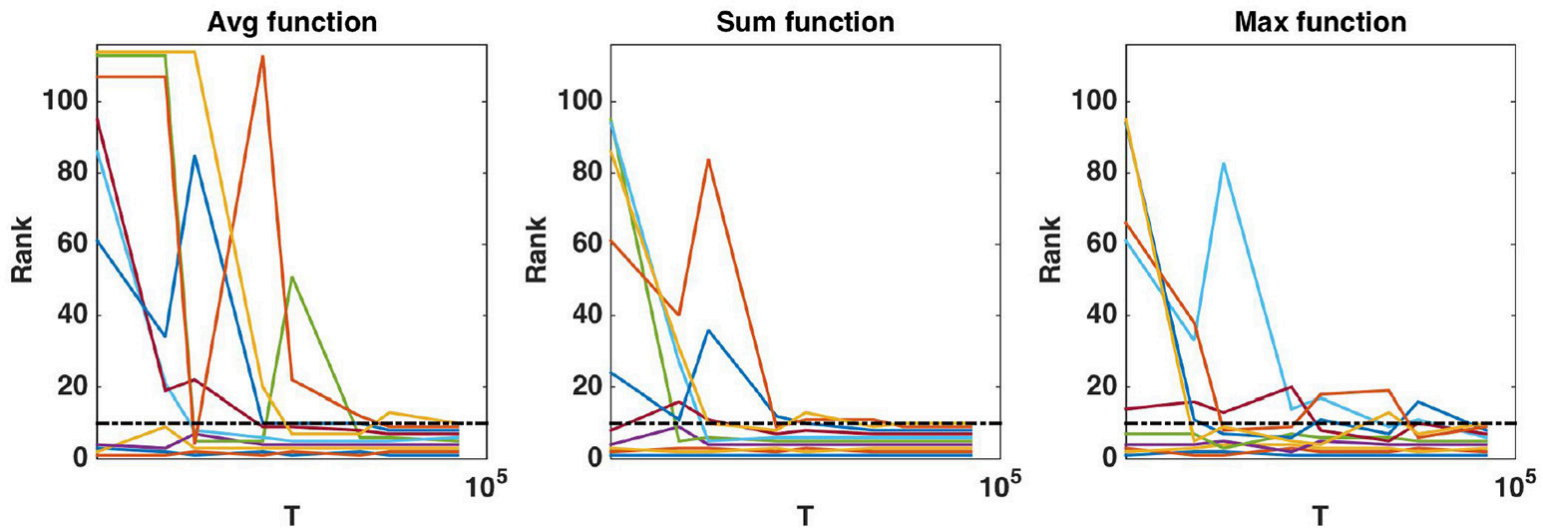
Real dataset. Evolution of the rank as a function of *T* parameter value (in log scale) for the first ten regions obtained with *T* = 10, 000 and $K=\sqrt{p}$. Importance scores are computed for the AAL atlas and for each aggregation function. Black horizontal dotted line represents the 10*th* ranking position.

While default values perform well in terms of error rate, it is interesting to study the impact of these parameters also on the stability of the group rankings. Using the AAL atlas, Figure 8 plots the evolution of the rank of ten groups when *K* is increased from 1 to *p* (and *T* is set to 10, 000), for the three aggregation functions. The ten groups are selected as the 10 most important groups when *K* = *p*, so that their rank converges towards {1, 2, …, 10} when *K* grows to *p*. The top four groups seem to remain the same whatever the value of *K*, as soon as *K* is not too small. The evolution of the rank of the other groups is however more chaotic, whatever the aggregation function, and some groups only reach the top ten when *K* is very close to *p*. Figure 9 shows the effect of *T* on the ranking of the top ten groups obtained with $K=\sqrt{p}$ and *T* = 10, 000. The number of trees has clearly a strong impact on rankings. Only the top 2 or 3 groups are already at their final position when *T* is small. The *sum* aggregation converges faster than the other two and it is the only one to have its top 10 groups fixed for *T* < 10, 000. As already shown by Huynh-Thu et al. (2012), this suggests that more trees are required to stabilise feature importances than to reach optimal predictive performance.

To compare and analyse further the different aggregation functions, Figure S3 shows the group importances and the individual voxel importances within each group for the top five groups ranked by the three aggregation functions (with $K=\sqrt{p}$ and *T* = 10, 000). The first four groups found by all aggregation functions are the same, while each function highlights a different group at the fifth position. The order between the top four groups however differs between functions but these differences can be explained. For example, the *sum* function puts group 85, which is larger, in front group 66, while they are ordered inversely with the *max* and *average* that are less sensitive to group sizes. While the maximum importance in group 85 is higher than the maximum importance in group 62, the *average* function prefers group 62 over group 85 because group 62 has less voxels of small or zero importance proportionally to its size.

**Figure 10:**
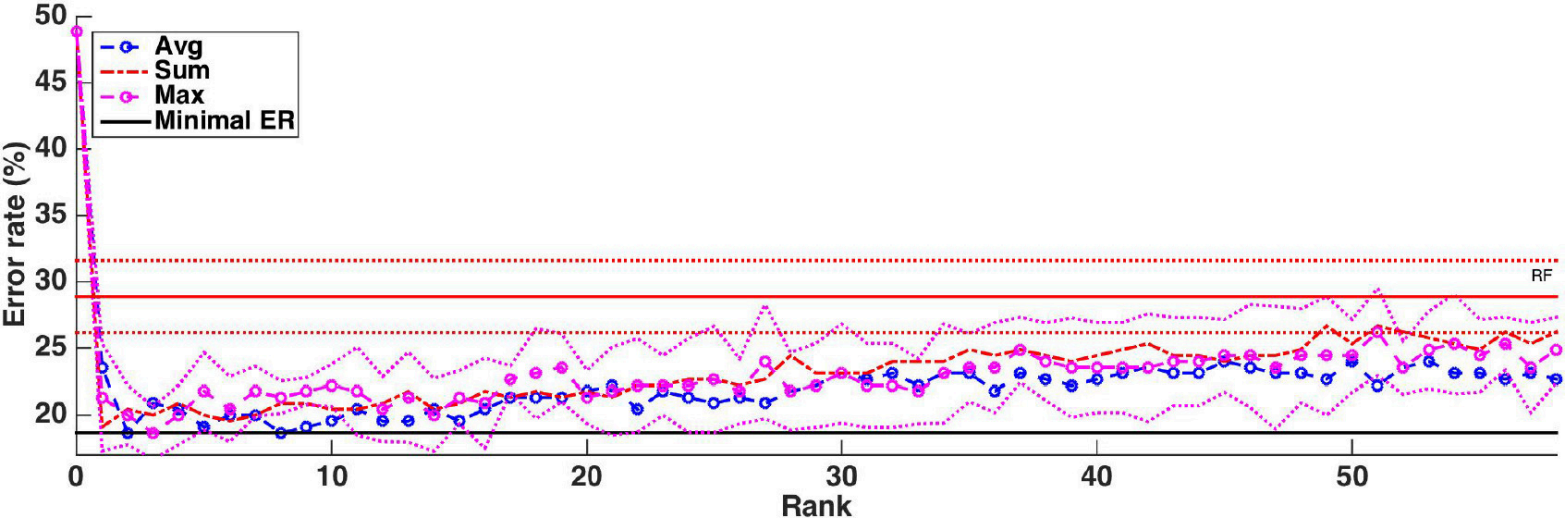
Real dataset. Error rates of a Random Forests classifier as a function of the number of groups included in the model (T =1,000 and $K=\sqrt{p}$). Errors are evaluated with a five repeated ten fold cross validation procedure. Error rate obtained with Random Forests (T=1,000 and $K=\sqrt{p}$) is represented by a red horizontal line, while the minimum error rate is represented by the black horizontal line. Standard deviations for RF and the *average* function are represented as dotted lines.

Without knowledge of the truly relevant groups, we can not assess group rankings using the AUPR, like we did on the artificial datasets. One common indirect way to evaluate a ranking is to build models using the top ranked features and see how it improves error rates: the better the ranking, the faster the error decreases when groups are introduced in the model. Figure 10 shows how the cross-validation error evolves when we progressively introduce the groups in the model following the rankings obtained with the three aggregation functions. The value 0 corresponds to a model always predicting the majority class without using any features. Errors were estimated as the average over five repeated ten-fold cross-validation runs. To avoid any selection bias in the evaluation, the groups are reranked at each iteration of each 10-fold cross-validation run without using the test fold. For comparison, we also show on the same plot the error obtained by Random Forests trained using all voxels (about 28%). One can see from this plot that it is possible to decrease the error rate from 28% (when using all voxels) to about 20% whatever the aggregation function used, suggesting that all group rankings contain informative groups at their top. This is consistent with results in Figure S3 that show that the top of the rankings are similar. The minimal error is reached in the three cases with a very small number of groups (respectively 8, 2, and 3 groups for the *average*, the *sum*, and the *max* aggregation), but the position of this minimum is clearly very unstable and almost optimal performance is reached with only a couple of groups. With the *max* and *average* aggregations (resp. with *sum* aggregation), the improvement over RF with all voxels is statistically significant (according to a t-test with risk level 0.05) when from 1 to 4 (resp. 5) groups are selected.

As a baseline for the obtained error rates, we also compare Random Forests with the MKL method proposed in Schrouff et al. (2018) using the AAL atlas and setting its parameter with an internal cross-validation as explained in the Methods section. We obtain an error rate of 39.56% with MKL, which is worse than the 28.89% error rate obtained with Random Forests and default setting.

#### 4.2.2. Group selection methods

We analyse here the output of the different group selection methods. In Figure S1, we illustrate how the statistical scores change when going down in the ranking, for each method and aggregation function. Scores of importance aggregated with the *sum* show a faster decrease than with the other aggregating functions. Regarding the selection methods, mProbes and CER are clearly more conservative methods since their statistical scores rapidly increase in all cases. The behaviour of CER^*r*^ is more dependent on the aggregation function used. With the *sum*, it is nearly as restrictive as mProbes and CER. However, when combined with *average* or *max*, score evolution is much more progressive, even more than eFDR. These observations are consistent with results on the artificial problems.

Table 2 summarizes the number of groups selected by each method (with α = 0.05) with every aggregation functions and different RF parameter settings. Overall, we observe very sparse results, with only a few, if any, groups selected in most settings. This is not surprising given the small size of the dataset and observations in the previous section (that show that an optimal error rate can be achieved with only a couple of groups). The only exception is the CER^*r*^ method which selects more groups with the *average* and *max* aggregation. We know however from experiments on the artificial data that this method has a low precision. In general, the *max* and *average* aggregation functions lead to the selection of more groups than the *sum*. Overall, with *K* = 1, increasing the number of trees from 1,000 to 10, 000 increases the number of selected groups. With $K=\sqrt{p}$, increasing *T* does not seem to affect the number of selected groups however. Comparing *K* = 1 and T=10,000 with $K=\sqrt{p}$ and T=1,000, we see that the latter setting leads to more groups overall, in particular when the mProbes method is used (it does not select any group with the *average* and *max* aggregation when *K* = 1). This suggests to set $K=\sqrt{p}$ and T≥1,000 to maximize the number of groups selected. Note however that this advise should be taken with caution since *K* could also affect the proportion of false positives among the selected groups.

**Table 2:** Number of regions selected (α = 0.05) for the real dataset for each method with the AAL atlas, depending on the aggregation function.

***(K;T)***	**CER**			**CER**^*****r*****^			**eFDR**			**mProbes**		
	**avg**	**∑**	**max**	**avg**	**∑**	**max**	**avg**	**∑**	**max**	**avg**	**∑**	**max**
(1;1,000)	0	2	1	9	0	2	0	2	1	0	1	0
(1;10,000)	0	2	3	10	0	8	0	2	3	0	2	0
($\sqrt{p}$;1,000)	2	3	2	17	1	8	0	3	3	2	3	1
($\sqrt{p}$;10,000)	0	3	2	>4	1	>4	0	4	4	2	5	3

#### 4.2.3. Interpretability

In this section, we analyse more precisely the groups selected with our methods and discuss them in the light of existing literature about MCI prognosis.

Several studies have looked at brain regions that impact AD prognosis. In univariate studies about AD prodromal stages, differences between MCI converters and non-converters have been identified to be localised mainly in the right temporoparietal and in the medial frontal area (Chételat et al., 2003, 2005; Drzezga et al., 2003; Nielsen et al., 2017). More precisely, according to the regions defined by the AAL atlas, the regions that are the most often identified as relevant for AD conversion are the superior temporal, the inferior parietal and the superior medial frontal. Several publications have also highlighted the middle temporal gyrus (right and left hemispheres) and the right angular gyrus (Morbelli et al., 2010). There thus only exist few regions discriminating converters and non-converters. Moreover, it remains a difficult task to differentiate these two classes of MCI as observed differences are generally very subtle. We believe this is consistent with the fact that most group selection methods only can find few regions.

It remains to be checked whether the regions found belong to the ones mentioned in the literature. For this purpose, we list in Table 3 the first ten top-ranked regions for all aggregation functions and for all RF parameter settings. With the *average* aggregation, brain regions at the first five positions vary a lot depending on the parameters *T* and *K*. Rankings are more stable with the *sum* and *max* aggregation functions. Overall, regions highlighted as the most important by all of these rankings are mostly consistent with studies about MCI progression towards Alzheimer's disease.

**Table 3:** Real dataset. First ten regions of rankings provided by Random Forests with different aggregation functions depending on parameters *K* and *T*.

	**(*K*; *T*) = (1;1, 000)**	**(*K*; *T*) = (1;10, 000)**	**$\left(K;T\right)=\left(\sqrt{p};1,000\right)$**	**$\left(K;T\right)=\left(\sqrt{p};10,000\right)$**
*avg*	Cuneus c. (L)	Angular g. (R)	Angular g. (R)	Middle temporal g. (R)
	Angular g. (R)	Middle temporal g. (R)	Middle temporal g. (R)	Angular g. (R)
	Middle temporal g. (R)	Vermic lob. 8	Inf. parietal (R)	Inf. parietal (R)
	Inf. parietal (R)	Vermic lob. 7	Middle temporal g. (L)	Middle temporal g. (L)
	Cerebelum 7b (R)	Middle temporal g. (L)	Thalamus (L)	Vermic lob. 7
	Inf. temporal g. (R)	Inf. parietal (R)	Cuneus c. (L)	Inf. temporal g. (R)
	Middle temporal g. (L)	Vermic lob. 6	Vermic lob. 8	Cuneus c. (L)
	Inf. temporal g. (L)	Inf. temporal g. (R)	Sup. temporal g. (R)	Inf. temporal g. (L)
	Sup. occipital g. (L)	Cuneus c. (L)	Heschl (R)	Sup. temporal g. (R)
	Olfactory (L)	Inf. temporal g. (L)	Inf. temporal g. (R)	Vermic lob. 8
∑	Middle temporal g. (L)	Middle temporal g. (L)	Middle temporal g. (R)	Middle temporal g. (R)
	Middle temporal g. (R)	Middle temporal g. (R)	Middle temporal g. (L)	Middle temporal g. (L)
	Inf. temporal g. (R)	Middle frontal g. (L)	Angular g. (R)	Angular g. (R)
	Inf. temporal g. (L)	Inf. temporal g. (R)	Inf. parietal (R)	Inf. parietal (R)
	Middle frontal g. (L)	Inf. temporal g. (L)	Inf. temporal g. (R)	Inf. temporal g. (R)
	Middle occipital g. (L)	Middle frontal g. (R)	Sup. temporal g. (R)	Inf. temporal g. (L)
	Precuneus (R)	Middle occipital g. (L)	Inf. temporal g. (L)	Sup. temporal g. (R)
	Middle frontal g. (R)	Sup. frontal g. (L)	Sup. temporal g. (L)	Cuneus c. (L)
	Cuneus c. (L)	PreCuneus c. (L)	Cuneus c. (L)	Sup. temporal g. (L)
	Sup. frontal g. (R)	Sup. temporal g. (R)	Cerebelum 6 (L)	Cerebelum 6 (R)
max	Middle temporal g. (R)	Middle temporal g. (L)	Middle temporal g. (R)	Middle temporal g. (R)
	Calcarine (R)	Sup. temporal g. (R)	Middle temporal g. (L)	Angular g. (R)
	Middle temporal g. (L)	Middle temporal g. (R)	Angular g. (R)	Middle temporal g. (L)
	Inf. temporal g. (R)	Inf. temporal g. (R)	Sup. temporal g. (R)	Inf. parietal (R)
	Angular g. (R)	Inf. temporal g. (L)	Inf. parietal (R)	Sup. temporal g. (R)
	Cuneus c. (L)	Angular g. (R)	PreCuneus c. (L)	Inf. temporal g. (R)
	Inf. parietal (L)	Hippocampus (R)	Calcarine (L)	Cerebelum 8 (L)
	Inf. frontal g. △ (L)	Thalamus (L)	Cuneus c. (L)	Cerebelum 6 (L)
	Inf. temporal g. (L)	Calcarine (L)	Inf. temporal g. (R)	Middle occipital g. (R)
	Postcentral g. (R)	Inf. occipital g. (L)	Temporal pole (Mid. temp. g. L)	Thalamus (L)

*R and L stand for right and left hemispheres respectively, g., gyrus; c., cortex; sup., superior; inf., inferior, △ denotes triangular part of the inferior frontal gyrus*.

**Figure 11:**
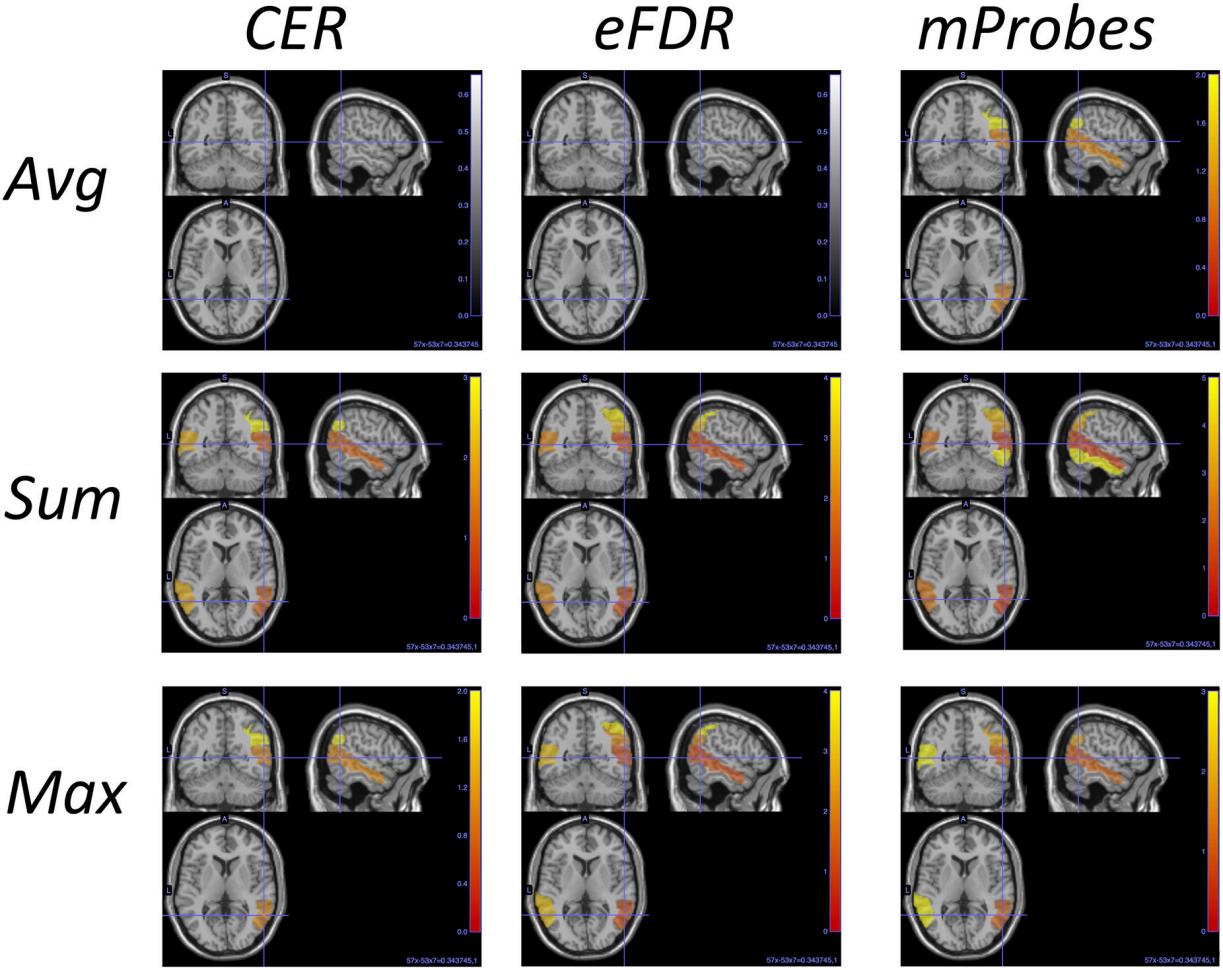
AAL regions selected with each method and each aggregation function for $K=\sqrt{p}$ and *T* = 10, 000. This picture is a visual representation of Table 4. The blob color provides information about the ranking: the more red the region is the better is its rank.

Table 3 can also be analysed along with the lines corresponding to the AAL atlas in Table 2 that show how many groups are considered as relevant by each selection method. To illustrate such analysis, we report in Table 4 for the top ranked AAL regions with the three aggregation functions the statistical scores estimated by CER, eFDR, and mProbes (with $K=\sqrt{p}$ and *T* = 10, 000). In each column, we only report the statistical scores until the first score higher than α = 0.05 (as next groups will be considered irrelevant anyway). We also provide a visual representation of this table in the brain space in Figure 11. Two groups are systematically selected as relevant (except by CER and eFDR with the *average* aggregation). These are the angular gyrus (right) and the middle temporal gyrus (right). With the *sum* and the *max* aggregations, eFDR and mProbes both select two additional regions: the middle temporal gyrus (left) and the inferior parietal (right). Finally, only mProbes selects the inferior temporal gyrus (right) with the *max* aggregation. These five regions are very consistent with the regions highlighted in the literature, as regions related to parietal and temporal areas are those that came out the most frequently.

**Table 4:** Real dataset. First top-ranked regions and corresponding statistical scores for different aggregation functions with $K=\sqrt{p}$ and *T* = 10, 000.

	**Regions**	**CER**	**eFDR**	**mProbes**
*avg*	Middle temporal g. (R)	0.057	0.057	0.046
	Angular g. (R)			0.042
	Inf. parietal (R)			0.215
∑	Middle temporal g. (R)	0	0	0.001
	Middle temporal g. (L)	0.006	0.003	0.013
	Angular g. (R)	0.006	0.003	0.020
	Inf. parietal (R)	0.081	0.030	0.042
	Inf. temporal g. (R)		0.051	0.046
	Inf. temporal g. (L)			0.065
max	Middle temporal g. (R)	0.010	0.010	0.003
	Angular g. (R)	0.028	0.016	0.019
	Middle temporal g. (L)	0.060	0.023	0.049
	Inf. parietal (R)		0.026	0.206
	Sup. temporal g. (R)		0.136	

*R and L stand for right and left hemisphere respectively, g., gyrus; sup., superior; inf., inferior*.

In comparison, averaging weights obtained over folds with MKL highlights the following regions in its top ten (in decreasing order of the weights): the middle temporal gyrus (right), the angular gyrus (right), the vermis 6 lobule, the thalamus (left), the frontal superior medial gyrus (right), the middle temporal gyrus (left), the vermis 8 lobule, the cerebelum 10 (left), the superior parietal gyrus (right) and the hippocampus (right). Regions selected are visually represented in the brain space in Figure S2. Although there are actually 76 regions over 116 with a non zero weight, we can however analyse how these weights are distributed. The first ranked region has a weight of 30 while the nine others show a weight between 9 and 2. After the tenth region, weights are slowly decreasing towards zero. The MKL top ten has three regions (out of five) in common with those highlighted with group selection methods, with two at the top of its ranking. Differences between the two lists are not unexpected given the different natures of the models (linear vs. non-parametric) and would deserve to be analysed more thoroughly.

## 5. Discussion

We proposed several methods based on Random Forests to select relevant groups of features on the basis of interpretable statistical scores. These methods are helpful in neuroimaging to improve the interpretability with respect to standard ML based analysis carried out at the level of voxels. In addition to an improvement of interpretability, group selection methods potentially exhibit a higher statistical power than feature selection methods. We have confirmed this through experiments on artificial datasets, where group methods are able to detect more relevant groups than similar methods working at the level of features. Moreover, on high dimensional datasets, computing statistical scores at the level of features can rapidly become very computational demanding. Working at the level of groups has thus only advantages when such groups naturally exist in the data.

We first assessed the behaviour of the different group selection methods through experiments on artificial problems where a group structure is imposed. By design, CER and mProbes are more conservative than eFDR and CER^*r*^. In terms of interpretability, CER^*r*^ is less reliable because it selects in general too many groups that can include a significant number of false positives. The other methods appear to be safe overall as they do not wrongly declare irrelevant groups as relevant. The comparison of the different aggregation functions to derive group importances from feature importances has shown that the *average* provides the best results, followed by the *max* and then the *sum*. The *sum* should be used carefully with *K* = 1 when groups of very different sizes are present in the data. Interestingly, when combined with group selection methods, this problem can however be diagnosed without knowledge of the truly relevant groups, as it will lead to no group being selected as relevant by any group selection method. Concerning the Random Forests parameters, $K=\sqrt{p}$ appears to detect more relevant groups than *K* = 1, although this latter setting has been shown theoretically to not suffer from masking effects.

We then applied the methods on a dataset related to Alzheimer's Disease prognosis. The conclusions are almost the same on this dataset, when methods are compared in terms of the number of groups they select. CER and mProbes are more conservative than eFDR and CER^*r*^. We thus recommend to use CER and mProbes to have more confidence in the selected regions. If reducing computing times is important, mProbes is clearly the best choice among these two as it only requires one round of permutations. Note however that all methods can be easily parallelised and in general, we believe that computing times should not really be an issue, especially when working with groups. As on the artificial datasets, using $K=\sqrt{p}$ leads to more groups than *K* = 1, as does increasing the number of trees *T*, which should be taken larger than for optimising error rate alone. No strong conclusion can be drawn concerning the aggregation functions however, as the three functions lead to very similar results. In particular, taking the *sum* does not show the same pathological behaviour as on the artificial data and actually can lead to more selected groups (e.g., Table 4).

Concerning Alzheimer's Disease prognosis, results are encouraging although they deserve to be analysed more thoroughly. Error rates are acceptable in our opinion, especially taking into account the small size of the dataset. They can be furthermore reduced significantly by focusing on a couple of groups. The group selection methods have highlighted several regions, e.g., the middle temporal gyrus (right) and the angular gyrus (right), that are consistent with the literature on MCI progression towards AD.

As future work, we would like to confirm our results on additional real datasets. While we focus here on interpretability, we would like also to explore more the possibility to improve predictive performance through group selection. Figure 10 shows that selecting a few groups can lead to improved error rates and in (Wehenkel et al., 2017), we showed that building Random Forests on the top of groups selected by CER^*r*^ could also improve performance. In our work, we use groups only to post-process Random Forests importance scores, but did not change anything in the way forests are grown. It would be interesting to investigate ways to incorporate groups directly during the Random Forests training stage, as it is done for example in the MKL framework (Schrouff et al., 2018) or in sparse linear methods (Jenatton et al., 2012).

## Author contributions

MW, PG, and CP conceived and designed the experiments. MW and AS performed the experiments. MW, CB, AS, PG, and CP analysed the results. CB, MW, and AS contributed materials. MW, CB, AS, PG, and CP wrote the paper.

### Conflict of interest statement

The authors declare that the research was conducted in the absence of any commercial or financial relationships that could be construed as a potential conflict of interest.
