# Supplementary material for: Random Forests Based Group Importance Scores and Their Statistical Interpretation: Application for Alzheimer's Disease
Source: Front Neurosci. 2018 Jun 29;12:411. doi: 10.3389/fnins.2018.00411 (PMC6034092; doi:10.3389/fnins.2018.00411)
Supplement: Supplementary file 1 [file Data_Sheet_1.pdf]

Supplementary Material: Random Forest based group importance scores and their statistical interpretation: application for Alzheimer's disease

Marie Wehenkel *, **Christine Bastin**, **Antonio Sutera**, **Pierre Geurts** and **Christophe Phillips**

*Correspondence:
Marie Wehenkel:
m.wehenkel@ulg.ac.be

1 REAL DATASET

1.1 Tables

Table S1. Demographic details of the MCI PET images from CRC. μ and σ stand for average and standard deviation respectively.

	Sex			Age		
	#	M	F	μ	σ	Range
MCI	23	14	9	73.43	7.80	58-84
MCIc	22	12	10	75.64	4.61	67-82

Table S2. Atlas information about the number of features per group. μ and σ stand for the average and the standard deviation of the number of features per group in the atlas respectively.

Atlas	μ	σ	Range
AAL	1431.3	1047.6	47-4791

1.2 Figures

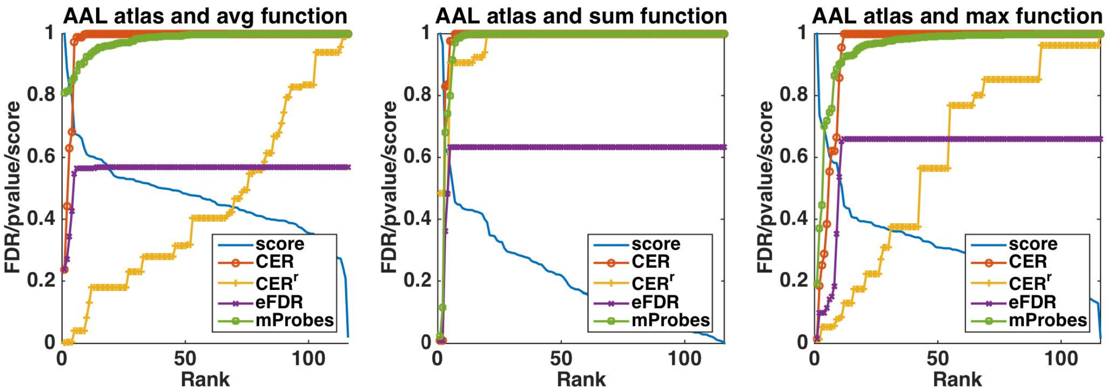

Figure S1: Curves of the importance scores and the different statistical scores obtained with the four methods for the AAL atlas and the CRC dataset. The curve labeled as 'Score' is the group importance score.

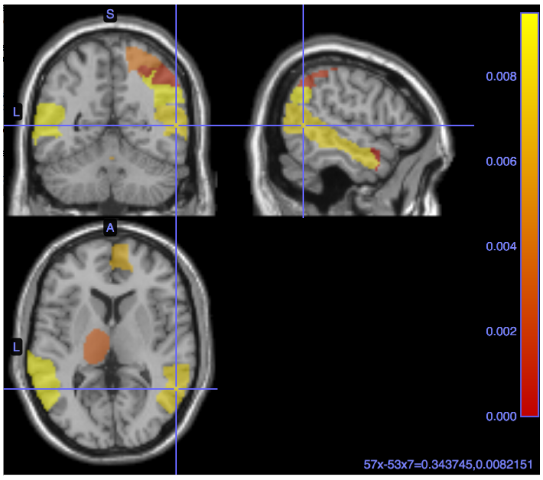

Figure S2: AAL regions selected with MKL. Weights are averaged over the ten repeated ten folds. The blob color provides information about the ranking: the more red the region is, the lower is its weight.

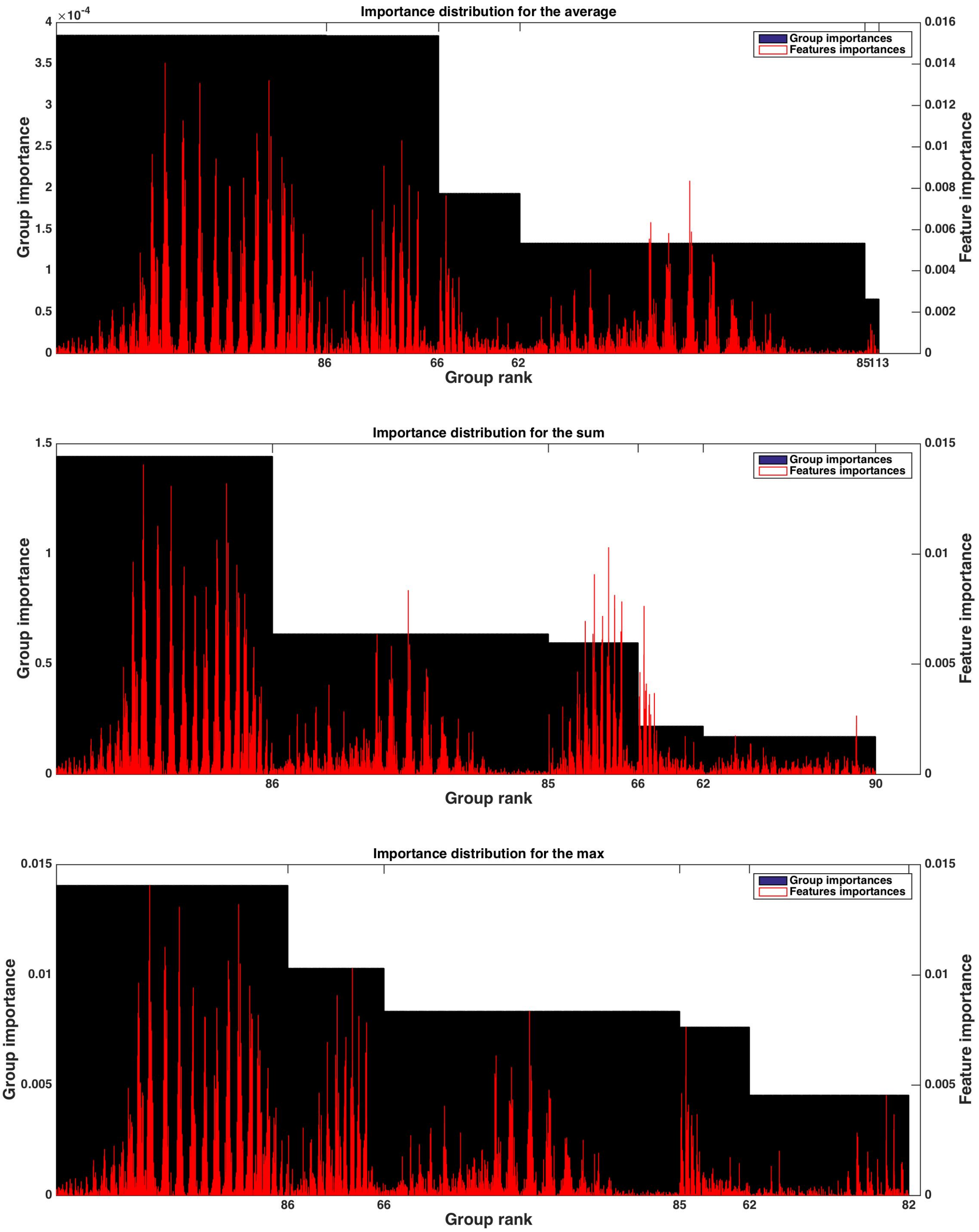

Figure S3: Group and individual voxel importances for the five groups of highest ranks, from top to bottom when using the *average*, *sum*, and *max* aggregation functions (with $K = \sqrt{p}$ and $T = 10,000$). X-axis shows the group number at the position of the last voxel within the group. Note that left y-axis is group importance, while right y-axis is voxel importance (different scales have been used for readibility).

2 DATA-DRIVEN ATLASES

To complement results in the paper with the AAL atlases, we provide in this section results with data-driven atlases. The motivation for data-driven atlases is that pre-defined atlases, such as AAL, are in general available only for the sake of result interpretation. They are typically used to label, in terms of structurally or functionally defined brain areas, the localization of the selected voxels but they are not necessarily representative of the group structure encoded by the data itself.

We consider here two clustering techniques to derive data-driven atlases: the hierarchical agglomerative clustering approach proposed in (Thirion et al., 2014) and an original hierarchical divisive approach inspired by regression tree construction methods. The idea of this technique is to learn a regression tree to predict the signal at each voxel of PET images from its 3D coordinates. The learning sample for growing this tree is thus composed of all $n \times p$ voxels measured in the learning sample described each by three input features corresponding to their x, y, and z coordinates and one numerical output corresponding to the signal at this voxel in the PET image. The leaves of the resulting regression tree will then define the disjoint groups of voxels of the atlas. The number of brain regions is set to a user-defined value k by limiting the maximum number of splits in the tree to $k - 1$ and by growing the tree using a best-first strategy (i.e., splitting at each step the leaf of highest output variance). Note that, since each tree split compares one coordinate with a threshold, the resulting groups will necessarily correspond to spatially connected brain regions as expected. A similar algorithm was exploited in (Geurts, 2001) to construct piecewise constant approximations of time series.

Both algorithms are unsupervised methods. They use information from the input matrix X to compose groups but have no concern for the labels Y.

Using these two algorithms, four atlases are derived:

- two atlases (denoted HC) obtained with hierarchical agglomerative clustering (Thirion et al., 2014), either with 116 regions, as in the AAL atlas, or with 1000 areas to test a finer resolution;
- two atlases (116 and 1000 regions) obtained with the divisive clustering approach described above, also with 116 and 1000 regions. We call this type of atlas "CART clustering".

Information about mean and standard deviation of the number of features per group for the four atlases is available in Table S3.

Similar experiments as with AAL atlas were reproduced with these four atlases. Table S4 shows the number of regions selected by each method and four Random forests parameter settings. These numbers follow similar trends as with the AAL atlas (in Table 2 of the main paper). Only very few regions are selected by all statistical scores, except CER^r which most probably suffer from a high false positive rate. Increasing the number of regions from 116 to 1000 does not necessarily increase the number of significant regions.

Analysing the groups selected with the data-driven atlases is more difficult as the corresponding brain regions have not been labelled. We attempted such analysis by looking at the AAL regions that overlap the groups ranked at the top for the data-driven atlases. The lists of these regions are reported in Tables S5, S6, S7 and S8 in the supplementary material respectively for the four data-driven atlases, CART_{116}, HC_{116}, CART_{1000}, and HC_{1000}. Lists are provided for each combination of aggregation function and Random Forests parameters. The number of top groups from the data-driven atlas that are projected on the AAL regions was determined for each aggregation function using the maximum between the number of groups

selected by CER and mProbes for this atlas with $K = \sqrt{p}$ and $T = 10,000$, as reported in the last part of Table S4. Using the same number of groups for all Random Forests parameter combinations allows to analyse the top ranking for the data-driven atlases even when no group is actually selected by CER and mProbes for this particular combination (for example, this is the case when $K = 1$ and $T = 1000$ with all atlases). From this information, we can thus assess whether the group selection methods were right not to select any group. To simplify the discussion, let us focus the analysis on the *average* aggregation (in the top parts of the tables). The interpretation of the results obtained with the *sum* and *max* aggregations is more difficult as these two functions often lead to no group selected or the selected groups correspond to too many groups from the AAL atlases to be analysed.

For the smaller atlases, CART_{116} and HC_{116}, the selected groups with $K = \sqrt{p}$, 4 for CART_{116} and 1 for HC_{116}, overlap with 21 groups from AAL for CART_{116} and 3 groups from AAL for HC_{116}. These groups do not depend on T and they contain several regions already highlighted earlier. When $K = 1$ with the same atlases, the AAL regions remains the same for HC_{116} although they are not selected any more by CER or mProbes when $T = 1000$. For CART_{116}, there are some differences in the AAL regions that are selected, although the AAL regions at the top of the ranking are very similar. Again, when $T = 1$, no regions are selected by CER and mProbes, suggesting that these methods are too conservative. With CART_{1000}, except for $(K = 1, T = 1000)$, 3 groups are selected by CER or mProbes that overlap with 5 or 6 AAL regions. These regions match very well the regions selected using the AAL atlas and are also very consistent with the literature. Two groups are selected in the case of HC_{1000} that leads to at most 4 groups from AAL that again contains regions highlighted in the literature (angular gyrus (left)) or when using the AAL atlas (parietal inferior (right)).

Overall, although the interpretation is less straightforward, results with the data-driven atlases and the average aggregation are consistent with the results obtained with the AAL atlas. The CART atlases seem also to better match the AAL atlas than the HC atlases.

2.1 Tables

Table S3. Atlas information about the number of features per group. μ and σ stand for the average and the standard deviation of the number of features per group in the atlas respectively.

Atlas	μ	σ	Range
HC_{116}	1894.2	2248.0	10-11,007
HC_{1000}	219.7	410.1	3-2712
$CART_{116}$	1894.2	1433.0	224-7121
$CART_{1000}$	219.7	188.33	18-1848

Table S4. Number of regions selected ($\alpha = 0.05$) for CRC dataset for each method and each atlas, depending on the aggregation function. HC and CART stand respectively for the use of the atlas obtained with hierarchical clustering and CART clustering.

$(K;T)$	Atlas	CER			CER^r			eFDR			mProbes		
		avg	$\sum$	max	*avg*	$\sum$	max	*avg*	$\sum$	max	avg	$\sum$	max
$(1;1000)$	HC_{116}	0	0	1	0	0	3	0	0	1	0	0	0
	$CART_{116}$	0	0	1	10	0	6	0	0	1	0	0	0
	HC_{1000}	0	0	1	9	0	4	0	0	2	0	0	0
	$CART_{1000}$	0	0	1	25	0	23	0	0	1	0	0	0
$(1;10,000)$	HC_{116}	1	0	0	0	0	2	1	0	0	0	0	0
	$CART_{116}$	2	0	4	20	0	8	2	0	4	0	0	0
	HC_{1000}	0	1	1	4	0	6	0	1	4	0	1	0
	$CART_{1000}$	3	0	2	29	0	32	5	0	6	0	0	0
$(\sqrt{p};1000)$	HC_{116}	1	0	0	0	0	3	1	0	0	1	0	0
	$CART_{116}$	4	3	1	17	6	10	4	5	6	3	1	1
	HC_{1000}	1	0	0	44	9	29	6	0	0	0	0	0
	$CART_{1000}$	3	2	4	92	17	39	16	7	10	3	2	2
$(\sqrt{p};10,000)$	HC_{116}	1	0	0	>1	0	>1	1	0	0	1	0	4
	$CART_{116}$	4	2	2	>5	>5	>5	4	5	5	2	2	1
	HC_{1000}	2	0	0	>9	6	>9	9	0	0	1	0	2
	$CART_{1000}$	3	2	4	>16	>16	>16	16	10	15	3	1	3

Table S5. CRC dataset. First top-ranked regions of the AAL atlas corresponding to the top-ranked regions of the $CART_{116}$ atlas selected with CER, $K = \sqrt{p}$ and $T = 10,000$, i.e. 4 region for the *avg*, 2 region for the *sum* and 2 regions for the *max*. Ranked are provided by Random Forest with different aggregation functions depending on parameters K and T. R and L stand for right and left hemisphere respectively.

	Rank	$(K;T) = (1;1,000)$	$(K;T) = (1;10,000)$	$(K;T) = (\sqrt{p};1,000)$	$(K;T) = (\sqrt{p};10,000)$
avg	1	Cerebelum Crus1 (R)	Cerebelum Crus1 (R)	Cerebelum Crus1 (R)	Cerebelum Crus1 (R)
	2	Inf. temporal g. (R)	Inf. temporal g. (R)	Inf. temporal g. (R)	Inf. temporal g. (R)
	3	Inf. occipital g. (R)	Inf. occipital g. (R)	Inf. occipital g. (R)	Inf. occipital g. (R)
	4	Mid. temporal g. (R)	Mid. temporal g. (R)	Mid. temporal g. (R)	Mid. temporal g. (R)
	5	Sup. temporal g. (R)	Sup. temporal g. (R)	Sup. temporal g. (R)	Sup. temporal g. (R)
	6	Angular g. (R)	Angular g. (R)	Angular g. (R)	Angular g. (R)
	7	Mid. occipital g. (R)	Mid. occipital g. (R)	Mid. occipital g. (R)	Mid. occipital g. (R)
	8	Parietal Inf (R)	Parietal Inf (R)	Parietal Inf (R)	Parietal Inf (R)
	9	Inf. temporal g. (L)	Fusiform (R)	SupraMarginal (R)	SupraMarginal (R)
	10	Mid. temporal g. (L)	Inf. temporal g. (L)	Postcentral (R)	Postcentral (R)
	11	Sup. temporal g. (L)	Mid. temporal g. (L)	Parietal Sup (R)	Parietal Sup (R)
	12	Rolandic Oper (L)	Sup. temporal g. (L)	Fusiform (R)	Fusiform (R)
	13	Heschl (L)	Rolandic Oper (L)	Inf. temporal g. (L)	Inf. temporal g. (L)
	14	Postcentral (L)	Heschl (L)	Mid. temporal g. (L)	Mid. temporal g. (L)
	15	SupraMarginal (L)	Postcentral (L)	Sup. temporal g. (L)	Sup. temporal g. (L)
	16	+ 10 others	+ 6 others	+ 6 others	+ 6 others
$\sum$	1	Frontal Inf Orb (R)	Frontal Inf Orb (R)	Temporal Mid (R)	Temporal Mid (R)
	2	Frontal Mid Orb (R)	Frontal Mid Orb (R)	Temporal Inf (R)	Temporal Inf (R)
	3	Frontal Sup Orb (R)	Frontal Sup Orb (R)	Temporal Sup (R)	Temporal Sup (R)
	4	Rectus (R)	Rectus (R)	Angular g. (R)	Angular g. (R)
	5	Rectus (L)	Rectus (L)	SupraMarginal (R)	SupraMarginal (R)
	6	Frontal Sup Orb (L)	Frontal Sup Orb (L)	Parietal Inf (R)	Parietal Inf (R)
	7	Frontal Mid Orb (L)	Frontal Mid Orb (L)	Postcentral (R)	Postcentral (R)
	8	Frontal Inf Orb (L)	Frontal Inf Orb (L)	Parietal Sup (R)	Parietal Sup (R)
	9	Frontal Mid Orb (R)	Frontal Mid Orb (R)	Temporal Inf (L)	Temporal Inf (L)
	10	Frontal Mid Orb (L)	Frontal Mid Orb (L)	Temporal Mid (L)	Temporal Mid (L)
	11	Cingulum Ant (L)	Cingulum Ant (L)	Temporal Sup (L)	Temporal Sup (L)
	12	Cingulum Ant (R)	Cingulum Ant (R)	Rolandic Oper (L)	Rolandic Oper (L)
	13	Frontal Mid (R)	Frontal Mid (R)	Heschl (L)	Heschl (L)
	14	Frontal Sup Medial (L)	Frontal Sup Medial (L)	Postcentral (L)	Postcentral (L)
	15	Frontal Sup Medial (R)	Frontal Sup Medial (R)	SupraMarginal (L)	SupraMarginal (L)
	16	+ 19 others	+ 21 others	+ 2 others	+ 2 others
max	1	Temporal Mid (R)	Temporal Inf (L)	Temporal Mid (R)	Temporal Mid (R)
	2	Temporal Inf (R)	Temporal Mid (L)	Temporal Inf (R)	Temporal Inf (R)
	3	Temporal Sup (R)	Temporal Sup (L)	Temporal Sup (R)	Temporal Sup (R)
	4	Angular g. (R)	Rolandic Oper (L)	Angular g. (R)	Angular g. (R)
	5	SupraMarginal (R)	Heschl (L)	SupraMarginal (R)	SupraMarginal (R)
	6	Parietal Inf (R)	Postcentral (L)	Parietal Inf (R)	Parietal Inf (R)
	7	Postcentral (R)	SupraMarginal (L)	Postcentral (R)	Postcentral (R)
	8	Parietal Sup (R)	Angular g. (L)	Parietal Sup (R)	Parietal Sup (R)
	9	Cerebelum Crus1 (L)	Precentral (L)	Temporal Inf (L)	Cerebelum Crus1 (R)
	10	Cerebelum Crus1 (R)	Temporal Mid (R)	Temporal Mid (L)	Cerebelum 6 (R)
	11	Lingual (L)	Temporal Inf (R)	Temporal Sup (L)	Fusiform (R)
	12	Lingual (R)	Temporal Sup (R)	Rolandic Oper (L)	Occipital Inf (R)
	13	Calcarine (L)	Angular g. (R)	Heschl (L)	Occipital Mid (R)
	14	Occipital Inf (R)	SupraMarginal (R)	Postcentral (L)	Calcarine (R)
	15	Occipital Inf (L)	Parietal Inf (R)	SupraMarginal (L)	Occipital Sup (R)
	16	+ 7 others	+ 2 others	+ 2 others	+ 0 others

Table S6. CRC dataset. First top-ranked regions of the AAL atlas corresponding to the top-ranked regions of the HC_{116} atlas selected with mProbes, $K = \sqrt{p}$ and $T = 10,000$, i.e. 1 region for the *avg*, 0 region for the *sum* and 4 regions for the *max*. Ranked are provided by Random Forest with different aggregation functions depending on parameters K and T. R and L stand for right and left hemisphere respectively.

	Rank	$(K;T) = (1;1,000)$	$(K;T) = (1;10,000)$	$(K;T) = (\sqrt{p};1,000)$	$(K;T) = (\sqrt{p};10,000)$
avg	1	Mid. occipital g. (L)	Mid. occipital g. (L)	Mid. occipital g. (L)	Mid. occipital g. (L)
	2	Angular g. (L)	Angular g. (L)	Angular g. (L)	Angular g. (L)
	3	Angular g. (R)	Angular g. (R)	Angular g. (R)	Angular g. (R)
$\sum$					
max	1	Frontal Sup Orb (R)	Cerebelum Crus1 (L)	Frontal Inf Orb (L)	Frontal Inf Orb (L)
	2	Fusiform (L)	Cerebelum Crus1 (R)	Frontal Inf Orb (R)	Frontal Inf Orb (R)
	3	Lingual (L)	Cerebelum Crus2 (L)	Frontal Mid Orb (R)	Frontal Mid Orb (L)
	4	Lingual (R)	Cerebelum 6 (R)	Mid. temporal g. (L)	Frontal Mid Orb (R)
	5	Cerebelum 6 (R)	Vermis 7	Amygdala (L)	Sup. temporal g. (R)
	6	Vermis 6	Vermis 6	Amygdala (R)	Insula (L)
	7	Cerebelum 6 (L)	Cerebelum 6 (L)	Sup. temporal g. (L)	Sup. temporal g. (L)
	8	Rectus (R)	Inf. temporal g. (R)	Insula (L)	Temporal Pole Sup (L)
	9	Rectus (L)	Inf. temporal g. (L)	Olfactory (R)	Temporal Pole Sup (R)
	10	Frontal Sup Orb (L)	Fusiform (R)	Olfactory (L)	Mid. temporal g. (L)
	11	Frontal Inf Orb (R)	Fusiform (L)	Temporal Pole Sup (L)	Insula (R)
	12	Frontal Inf Orb (L)	Cerebelum 3 (R)	Temporal Pole Sup (R)	Hippocampus (L)
	13	Frontal Mid Orb (L)	Vermis 3	Hippocampus (R)	Caudate (R)
	14	Frontal Mid Orb (R)	Cerebelum 3 (L)	Hippocampus (L)	Caudate (L)
	15	Olfactory (R)	Vermis 1 2	Sup. temporal g. (R)	Olfactory (R)
	16	+ 86 others	+ 80 others	+ 80 others	+ 68 others

Table S7. CRC dataset. First top-ranked regions of the AAL atlas corresponding to the top-ranked regions of the CART_{1000} atlas selected with CER, $K = \sqrt{p}$ and $T = 10,000$, i.e. 3 regions for the *avg*, 2 regions for the *sum* and 4 regions for the *max*. Ranked are provided by Random Forest with different aggregation functions depending on parameters K and T. R and L stand for right and left hemisphere respectively.

	Rank	$(K;T) = (1;1,000)$	$(K;T) = (1;10,000)$	$(K;T) = (\sqrt{p};1,000)$	$(K;T) = (\sqrt{p};10,000)$
avg	1	Inf. temporal g. (L)	Mid. temporal g. (R)	Mid. temporal g. (R)	Mid. temporal g. (R)
	2	Mid. temporal g. (L)	Sup. temporal g. (R)	Sup. temporal g. (R)	Sup. temporal g. (R)
	3	Insula (R)	Angular g. (R)	Angular g. (R)	Angular g. (R)
	4	Frontal Inf Tri (R)	SupraMarginal (R)	SupraMarginal (R)	SupraMarginal (R)
	5	Frontal Mid Orb (L)	Inf. temporal g. (R)	Parietal Inf (R)	Parietal Inf (R)
	6	Frontal Inf Orb (L)	Parietal Inf (R)		
$\sum$	1	Calcarine (R)	Calcarine (R)	Mid. temporal g. (R)	Mid. temporal g. (R)
	2	Lingual (R)	Lingual (R)	Sup. temporal g. (R)	Sup. temporal g. (R)
	3	Lingual (L)	Lingual (L)	Angular g. (R)	Angular g. (R)
	4	Calcarine (L)	Calcarine (L)	SupraMarginal (R)	SupraMarginal (R)
	5	Precuneus (L)	Precuneus (L)		
	6	Cuneus (R)	Cuneus (R)		
	7	Cuneus (L)	Cuneus (L)		
	8	Precuneus (R)	Precuneus (R)		
	9	Cingulum Mid (R)	Cingulum Mid (R)		
	10	Cingulum Mid (L)	Cingulum Mid (L)		
	11	Frontal Sup Medial (L)	Frontal Sup Medial (L)		
	12	Frontal Sup Medial (R)	Frontal Sup Medial (R)		
	13	Supp Motor Area (L)	Supp Motor Area (L)		
	14	Supp Motor Area (R)	Supp Motor Area (R)		
	15	Frontal Sup (R)	Frontal Sup (R)		
max	1	Mid. temporal g. (R)	Inf. temporal g. (L)	Mid. temporal g. (R)	Mid. temporal g. (R)
	2	Sup. temporal g. (R)	Mid. temporal g. (L)	Sup. temporal g. (R)	Sup. temporal g. (R)
	3	SupraMarginal (R)	Sup. temporal g. (L)	SupraMarginal (R)	SupraMarginal (R)
	4	Lingual (R)	Heschl (L)	Angular g. (R)	Angular g. (R)
	5	Calcarine (L)	Rolandic Oper (L)	Inf. temporal g. (L)	Inf. temporal g. (R)
	6	Lingual (L)	Postcentral (L)	Mid. temporal g. (L)	Mid. occipital g. (R)
	7	Inf. occipital g. (L)	SupraMarginal (L)	Sup. temporal g. (L)	Parietal Inf (R)
	8	+ 13 others	+ 8 others	+ 6 others	+ 0 others

Table S8. CRC dataset. First top-ranked regions of the AAL atlas corresponding to the top-ranked regions of the HC_{1000} atlas selected with $K = \sqrt{p}$ and $T = 10,000$, i.e. 2 regions for the *avg* with CER, 0 region for the *sum* and 2 regions for the *max* with mProbes. Ranked are provided by Random Forest with different aggregation functions depending on parameters K and T. R and L stand for right and left hemisphere respectively.

	Rank	$(K;T) = (1;1,000)$	$(K;T) = (1;10,000)$	$(K;T) = (\sqrt{p};1,000)$	$(K;T) = (\sqrt{p};10,000)$
avg	1	Frontal Inf Orb (L)	Parietal Inf (L)	Angular g. (L)	Angular g. (L)
	2	Parietal Inf (L)	Parietal Inf (R)	Angular g. (R)	Angular g. (R)
	3		Angular g. (L)		Parietal Inf (R)
	4		Angular g. (R)		
$\sum$					
max	1	Frontal Inf Tri (R)	Inf. occipital g. (L)	Frontal Mid Orb (R)	Frontal Mid Orb (L)
	2	Pallidum (L)	Inf. occipital g. (R)	Frontal Inf Orb (L)	Frontal Inf Orb (L)
	3	Pallidum (R)	Calcarine (L)	Frontal Inf Orb (R)	Frontal Mid Orb (R)
	4	Thalamus (L)	Lingual (R)	Insula (R)	Frontal Inf Orb (R)
	5	Thalamus (R)	Fusiform (L)	Olfactory (R)	Sup. temporal g. (L)
	6	Vermis 4 5	Frontal Inf Orb (R)	Olfactory (L)	Temporal Pole Sup (L)
	7	Mid. temporal g. (R)	Frontal Inf Orb (L)	Caudate (L)	Temporal Pole Sup (R)
	8	Calcarine (L)	Frontal Mid Orb (R)	Cingulum Ant (L)	Insula (L)
	9	Mid. temporal g. (L)	Frontal Sup Orb (L)	Frontal Mid Orb (R)	Insula (R)
	10	Calcarine (R)	Frontal Mid Orb (L)	Cingulum Ant (R)	Caudate (R)
	11	Lingual (R)	Caudate (R)	Frontal Mid Orb (L)	Caudate (L)
	12	Occipital Sup (R)	Temporal Pole Sup (R)	Frontal Mid Orb (L)	Olfactory (R)
	13	Cuneus (R)	Olfactory (L)	Caudate (R)	Olfactory (L)
	14	Mid. occipital g. (R)	Caudate (L)	Putamen (R)	Lingual (L)
	15	Cingulum Ant (R)	Insula (R)	Mid. temporal g. (L)	ParaHippocampal (L)
	16	+ 26 others	+ 47 others	+ 46 others	+ 26 others

REFERENCES

Geurts, P. (2001). Pattern extraction for time series classification. In *Proceedings of the 5th European Conference on Principles of Data Mining and Knowledge Discovery* (London, UK, UK: Springer-Verlag), PKDD '01, 115–127

Thirion, B., Varoquaux, G., Dohmatob, E., and Poline, J.-B. (2014). Which fMRI clustering gives good brain parcellations? *Frontiers in neuroscience* 8
